# Protein import complexes in the mitochondrial outer membrane of Amoebozoa representatives

**DOI:** 10.1186/s12864-016-2402-2

**Published:** 2016-02-06

**Authors:** Dorota Buczek, Małgorzata Wojtkowska, Yutaka Suzuki, Seiji Sonobe, Yukinori Nishigami, Monika Antoniewicz, Hanna Kmita, Wojciech Makałowski

**Affiliations:** Institute of Bioinformatics, Faculty of Medicine, University of Muenster, Niels Stensen Strasse 14, 48149 Muenster, Germany; Laboratory of Bioenergetics, Institute of Molecular Biology and Biotechnology, Faculty of Biology, Adam Mickiewicz University, Umultowska 89, 61-614 Poznań, Poland; Department of Life Science, Graduate School of Life Science, University of Hyogo, Harima Science Park City, Kobe, Hyogo 678-1297 Japan; Department of Medical Genome Sciences, Graduate School of Frontier Sciences, The University of Tokyo, Kashiwa, Chiba 277-8562 Japan

**Keywords:** Amoebozoa, Protein import into mitochondria, Transcriptome analysis, TOM complex, TOB/SAM complex, ERMES complex

## Abstract

**Background:**

An ancestral trait of eukaryotic cells is the presence of mitochondria as an essential element for function and survival. Proper functioning of mitochondria depends on the import of nearly all proteins that is performed by complexes located in both mitochondrial membranes. The complexes have been proposed to contain subunits formed by proteins common to all eukaryotes and additional subunits regarded as lineage specific. Since Amoebozoa is poorly sampled for the complexes we investigated the outer membrane complexes, namely TOM, TOB/SAM and ERMES complexes, using available genome and transcriptome sequences, including transcriptomes assembled by us.

**Results:**

The results indicate differences in the organization of the Amoebozoa TOM, TOB/SAM and ERMES complexes, with the TOM complex appearing to be the most diverse. This is reflected by differences in the number of involved subunits and in similarities to the cognate proteins of representatives from different supergroups of eukaryotes.

**Conclusions:**

The obtained results clearly demonstrate structural variability/diversity of these complexes in the Amoebozoa lineage and the reduction of their complexity as compared with the same complexes of model organisms.

**Electronic supplementary material:**

The online version of this article (doi:10.1186/s12864-016-2402-2) contains supplementary material, which is available to authorized users.

## Background

Mitochondria are the cell’s “power house” and serve as a reservoir of factors, which reinforce signals for cell life and death. Therefore, mitochondria are vital for the majority of eukaryotes. Their construction is based on the outer and inner membranes, which form two internal aqueous compartments: the intermembrane space and matrix. During the evolution of a eukaryotic cell, mitochondria retained the genetic system of a prokaryotic ancestor in a form of mitochondrial DNA localized in mitochondrial matrix. However, this encodes only one to ten per cent of all mitochondrial proteins whereas the remainder mitochondrial proteins in present-day cells were left to be encoded by nuclear genes [1, 2]. These include proteins encoded by genes that were transferred from the prokaryotic endosymbiont to the nucleus, as well as by new genes that arose during eukaryotic evolution. Moreover, the import concerns all proteins of the mitochondrial outer membrane and the intermembrane space, as well as the majority of the inner membrane and matrix proteins (e.g. [3]).

Undoubtedly, the import of proteins into the mitochondria is crucial for the proper function of mitochondria and its implementation requires the formation of protein heterocomplexes in both mitochondrial membranes. The complexes, also defined as molecular machineries function as protein translocases importing proteins from the site of their synthesis in the cytosol to a given compartment of mitochondria (e.g. [1, 3–9]). At the mitochondrial outer membrane, the import is mediated by the TOM complex (translocase of the outer membrane) and the TOB/SAM complex (sorting and assembly machinery/topogenesis of the mitochondrial outer membrane β-barrel proteins). The TOM complex is regarded as a general gateway for mitochondria, as the complex is not only responsible for the translocation of imported proteins across or into the outer membrane but also for decoding their targeting signals and subsequent sorting. The TOB/SAM complex receives imported proteins from the TOM complex and performs their integration and assembly into the outer membrane, including β-barrel integral proteins and other subunits of the TOM complex. Moreover, the endoplasmic reticulum membrane (ER) is connected to the outer membrane by the ER-mitochondria encounter structure (ERMES complex) that tethers the ER to mitochondria by the interaction with the TOB/SAM complex. The ERMES complex is involved in a plethora of mitochondrial processes, including mitochondrial morphology and the assembly of β-barrel proteins into the mitochondrial outer membrane [10–13].

As recently summarized by Sokol et al. [14], extensive studies of mitochondrial protein import using the model yeast *Saccharomyces cerevisiae* uncovered the canonical components of different translocases and provided detailed mechanistic and topological information about their function and interplay. The TOM complex of *S. cerevisiae* contains a channel forming subunit (Tom40), receptors for various classes of incoming precursor proteins (Tom20 and Tom70), an internal receptor for mitochondrial precursor proteins which also maintains the complex architecture (Tom22), and modulators of the complex assembly and stability (Tom5, Tom6, and Tom7). The TOB/SAM complex in turn, consists of a channel forming subunit (Tob55/Sam50), which cooperates with Sam35/Tob38 and Sam37/Mas37 in the recognition, transport and integration of β-barrel proteins into the membrane. Additionally, the TOB/SAM complex associates with Mdm10, which is specifically required in late stage of the TOM complex assembly. Mdm10 is also crucial to the ERMES complex. In the complex, Mdm10 and Gem1 are connected with the help of Mdm12 and Mdm34 (termed also Mmm2) to the ER integral membrane protein Mmm1 [10, 15, 16].

Mitochondrial protein import complexes, including the TOM and TOB/SAM complexes, are of modular construction. This means that each complex contains subunits formed by proteins common to all eukaryotes and additional subunits that have been added over time and are regarded to be lineage specific [1, 17–20]. Available data indicate that the subunit organization of the TOM and TOB/SAM complexes of animals and plants is more or less similar to those described for *S. cerevisiae* [14, 21]. For example, besides Tob55/Sam50, the plant TOB/SAM complex contains Metaxin being an orthologue of Sam37/Mas37 [21] whereas in the case of human TOB/SAM complex, Metaxin-2 is an counterpart of Tob38/Sam35 while Metaxin-1 and Metaxin-3 are counterparts of Mas37/Sam37 [14]. Contrariwise, in the case of other eukaryotes the differences are more pronounced. Consequently, the commonly occurring subunits of the TOM and the TOB/SAM complexes are Tom40 and Tob55/Sam50, respectively, both forming channels of β-barrel topology [22–25]. Additionally, Tom7 is generally present in mitochondria of representative organisms from the major phylogenetic lineages, but the presence of other Tob/Sam and Tom proteins remains elusive. On the other hand, it is suggested that the ERMES complex subunits, with the exception of Gem1, occur concomitantly in representatives of different phylogenetic lineages. Accordingly, the ERMES complex subunits identified in *S. cerevisiae* have been shown to be lost in animals and plants but are present, in fungi, and in some protists such as slime molds classified as representatives of the Amoeoboza [12].

Given the polyphyletic character of protists, the historic division of eukaryotic organisms into four kingdoms of Plantae, Animalia, Fungi, and Protista has been replaced by a new system that consists of six large supergroups, namely Chromalveolata, Excavata, Archaeplastida, Rhizaria, Amoebozoa, and Opisthokonta [26–28]. The Amoebozoa is regarded as a sister clade to Opisthokonta, involving fungi and animals. Recent molecular studies divide the Amoebozoa into two major subclades, Lobosa and Conosa, with possibly a third lineage, Breviatea [29]. As summarized by Fiz-Palacios et al. [30], Lobosa is divided further into two subdivisions: Discosea and Tubulinea whereas Conosa is subdivided into three: Variosea, Archamoebea, and Mycetozoa or slime molds.

Since little investigation has been done on Amoebozoa mitochondrial protein import complexes, we investigated the complexes at the genome and transcriptome levels. Several genomes and transcriptomes of amoebozoans representing the different subclades and subdivisions of Amoebozoa were analyzed for subunit organization of the TOM, TOB/SAM and ERMES complexes. The obtained results indicate differences in organization of the Amoebozoa complexes, in regards to the number of involved subunits and their amino acid sequences. Moreover, despite the proposed close relationship between Opisthokonta and Amoebozoa, some of the predicted TOM subunits displayed the highest similarity to the cognate proteins of plants and Excavata. Furthermore, the predicted TOM complex subunits do not seem to support the proposed systematic division of the Amoebozoa, whereas the subunits of the TOB/SAM and ERMES complexes appear to confirm the division.

## Results

### Amoebozoans differ in organization of the TOM complex

The “orthodox” TOM complex of *Saccharomyces cerevisiae* consists of Tom70, Tom40, Tom22, Tom20, Tom7, Tom6 and Tom5 [14]. Search of the reference protein sequences (see Additional file 1: Table S1) against the proteome, transcriptome, and genome of *A. castellanii* resulted in finding five out of seven canonical subunits of the TOM complex, i.e. Tom7, Tom20, Tom22, Tom40 and Tom70 (Table 2). Most of the proteins were identical to proteins deposited in the GenBank, namely Tom7 (XP_004340925, 64 amino acids (AA)), Tom20 (XP_004333415, 273 AA), and Tom40 (ADZ24223 and XP_004337172, 361 AA). The sequence encoding Tom70 (907 AA) differed slightly from the sequence stored in GenBank under accession number XP_004339622 (898 AA) (Additional file 1: Figure S1A). Although the sequence of annotated gene XP_004353494 was identical to our predicted Tom22 (Tom22 A), we noticed a potential in-frame START codon 78 nucleotides upstream of the annotated coding DNA sequence (CDS) (Additional file 1: Figure S1B). Interestingly, the resulting extra peptide seems to be a signal peptide as suggested by SignalP analysis [31]. However, the signal sequence is different from the import signal of the model Tom22 identified for *Neurospora crassa* [32]. Intruigingly, there was a second copy of the Tom22 gene in the *A. castellanii* genome. As shown in Additional file 1: Figure S1C, it also appeared to contain 78 nucleotides upstream of the annotated CDS (XP_004358239). Although located on an “unplaced” scaffold, the gene was clearly distinct from Tom22 A encoding gene, and RNA-seq data demonstrated its expression. Interestingly, the open reading frame of the second gene resulted in a protein (Tom22 B) of amino acid content (158 AA) comparable to the canonical one, due to a frameshift resulting in a premature stop codon as compared to Tom22 A encoding gene. Although the number of synonymous changes between Tom22 A and Tom22 B encoding genes exceeds the number of nonsynonymous changes (d_N_/d_S_ = 0.69), both codon-based Fisher’s exact tests of selection and codon-based Z-test of selection (p = 1 and p = 0.574, respectively) suggest that the difference is not significant. Therefore, a neutrality hypothesis cannot be rejected. In the case of *A. proteus* the genome sequence is not available. Therefore, the transcriptome (AP_RNASeq) was used to perform similarity searches and three Tom proteins were found (Table 2); namely, Tom20 (281 AA), Tom40 (283 AA) and Tom70 (911 AA).

The available genomes (Table 1) and protein datasets were also used to perform similarity search analysis of the TOM complex subunits of *D. discoideum, D. purpureum, D. fasciculatum* and *P. pallidum*. For the slime molds, we found Tom7, Tom20, Tom40 and Tom70 (Table 2), although Tom7 was only predicted for *D. discoideum* (XP_639342, 55 AA) and *P. pallidum* (EFA78398, 54 AA). The identified sequences of Tom7 were in agreement with previously published data [33, 34]. Tom20 was detected for all the slime molds, i.e. for *D. discoideum* (XP_642375, 366 AA)*, D. fasciculatum* (XP_004357260, 330 AA)*, D. purpureum* (XP_003295156, 337 AA)*, P. pallidum* (EFA76591, 352 AA)*.* Likewise, Tom40 and Tom70 were found for all studied slime molds namely, for *D. discoideum* (Tom40: XP_642798, 314 AA; Tom70: XP_645222, 1050 AA), *D. fasciculatum* (Tom40: XP_004352318, 301 AA; Tom70: XP_004358771, 554 AA), *D. purpureum* (Tom40: XP_003286519, 312 AA; Tom70: XP_003283061, 1045 AA), *P. pallidum* (Tom40: EFA80126, 298 AA; Tom70: EFA81504, 763 AA). Tom40 of *D. discoideum*, *D. purpureum* and *P. pallidum* were identical to the proteins previously predicted by Wojtkowska et al. (2012) [35].

**Table 1:** Availability of genome and transcriptomes of studied amoebozoans

Accessibility of:	*A. c*	*A. p*	*D. d*	*D. f*	*D. p*	*P. p*	*E. d*	*E. n*
Genome	yes	no	yes	yes	yes	yes	yes	yes
Transcriptome	**yes**	**yes**	yes	no	yes	yes	no	no
GenBank project ID	66753, 193615	-	201, 13925	40189, 193617	30991, 63531	40191, 46447	28615, 12914	25730, 72167
Genome size [Mb]	42.02	293000^a^	34.21	31.02	32.97	32.97	30.63	14.40

Bold letters denote transcriptome sequences assembled by us for this study

^a^[60, 61]

**Table 2:**
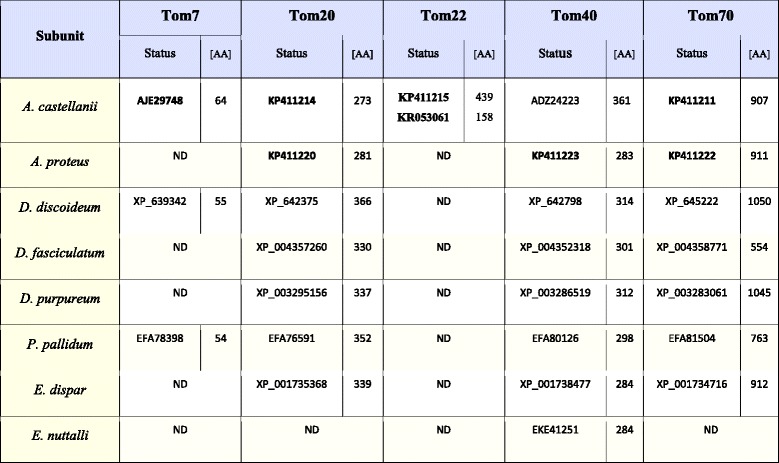
The identified subunits of the studied Amoebozoa TOM complex

ND indicates proteins not detected in all datasets. Bold numbers denote sequences determined in this study

In the case of *E. dispar* and *E. nuttalli,* the similarity searches were also performed against protein datasets as well as genomic data (Table 1). As shown in Table 2, Tom20 and Tom70 were found only in *E. dispar* (Tom20: XP_001735368, 339AA; Tom70: XP_001734716, 912 AA). Yet, Tom40 was found both for *E. nuttalli* (XP_001738477, 284 AA) and *E. dispar* (EKE41251, 284 AA). The latter was identical to the sequence previously detected by Wojtkowska et al. (2012) [35].

Summing up, the number of predicted subunits of the TOM complex varied among the studied amoebozoans. The highest number was observed for *A. castellani* (five subunits) and the lowest for *E. nuttalli* (one subunit). Accordingly, the TOM complex of *E. dispar* and *A. proteus* as well as *D. fasciculatum* and *D. purpureum* appeared to contain three subunits, whereas the complex of *D. discoideum* and *P. pallidum* were predicted to consist of four subunits. Importantly with the exception of *A. castellanii,* all complexes appeared to be depleted of Tom22.

### The studied amoebozoans differ in organization of the TOB/SAM complex

The canonical TOB/SAM complex of *S. cerevisiae* is reported to contain Tob55/Sam50 and two additional proteins called Tob38/Sam35 and Mas37/Sam37, regarded as counterparts of plant and mammalian Metaxins [14, 21]. Therefore, in addition to Tob38/Sam35 and Mas37/Sam37, we used Metaxins in BLAST searches. It is also known that the TOB/SAM complex interacts with Mdm10, which is a member of the ERMES complex (see below).

Similarity searches against AC_RNASeq (see Additional file 1: Table S1), combined with searches within the available protein datasets of *A. castellanii* as well as the genomic data (Table 1), resulted in the identification of Tob55/Sam50 and Metaxin (Table 3). The sequence encoding Metaxin was different from the sequence stored in GenBank under accession number XP_004337900 (275 AA). The Metaxin amino acid sequence based on our transcriptome data was longer (296 AA) and contained two additional insertions of 18 and 3 amino acids (Additional file 1: Figure S1D). An analogous difference was observed for *A. castellanii* Tob55/Sam50. The sequence stored in GenBank under accession number XP_004341043, (376 AA) differed from the protein revealed by AC_RNASeq analysis. The latter was longer by 21 residues (446 AA in total) and contained two additional insertions of 44 and 26 amino acids (Additional file 1: Figure S1E). As shown in Table 3, analysis of AP_RNASeq resulted in the identification of *A. proteus* Metaxin (294 AA) and Tob55/Sam50 (459 AA).

**Table 3:**
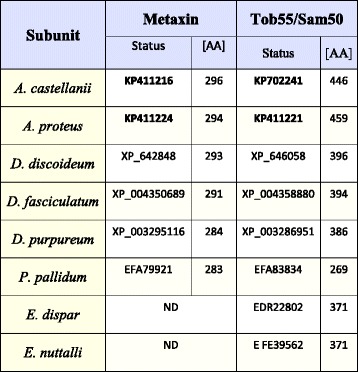
The identified subunits of the studied Amoebozoa TOB/SAM complex

ND indicates proteins not detected in all datasets. Bold numbers denote sequences determined in this study

Tob55/Sam50 and Metaxin were also identified for all studied slime molds (Table 3); namely, *D. discoideum* (Metaxin: XP_642848, 293 AA; Tob55/Sam50: XP_646058, 396 AA), *D. fasciculatum* (Metaxin: XP_004350689, 291 AA; Tob55/Sam50: XP_004358880, 394 AA), *D. purpureum* (Metaxin: XP_003295116, 284 AA; Tob55/Sam50: XP_003286951, 386 AA), *P. pallidum* (Metaxin: EFA79921, 283 AA; Tob55/Sam50: EFA83834, 296 AA). Tob55/Sam50 of *D. discoideum*, *D. purpureum*, and *P. pallidum* were identical with sequences previously predicted by Wojtkowska et al. [35].

In the case of *E. dispar* and *E. nuttalli* (Table 3), available protein datasets and genomic data analysis resulted only in the detection of Tob55/Sam50, EDR22802 (371 AA) and EKE39562 (371 AA), respectively. The sequence of *E. dispar* Tob55/Sam50 was identical to the sequence predicted by Wojtkowska et al. (2012) [35].

The obtained results indicated that in the case of the studied amoebozans, the organization of the TOB/SAM complex was similar and included Tob55/Sam50 and Metaxin. However, it should be noted that the *E. nuttalli* and *E. dispar* TOB/SAM complexes did not contain Metaxin.

### The studied amoebozoans differ in organization of the ERMES complex

The canonical ERMES complex of *S. cerevisiae* consist of Mdm10, Mdm12, Mdm34/Mmm2, Gem1 and Mmm1, the latter located in the ER membrane [11, 15, 16]. Similarity searches against AC_RNASeq (see Additional file 1: Table S1), combined with searches of available protein data sets of *A. castellanii* as well as the genomic data indicated the presence of all subunits of the ERMES complex; namely, Mdm10, Mdm12, Mdm34/Mmm2, Mmm1 and Gem1 (Table 4). The sequences predicted for Mdm10 (402 AA), Mdm12 (240 AA) and Mdm34/Mmm2 (245 AA) displayed no differences when compared to the sequences stored in GenBank under the accession numbers of XP_004352736, XP_004340225 and XP_004340163, respectively (Table 4). The sequence encoding Gem1 (610 AA) and based on transcriptome data differed from a sequence already stored in GenBank under the accession number of XP_004356731 (586 AA). The former contained additional 23 amino acids close to the N-terminus (Additional file 1: Figure S1F). Surprisingly, Mmm1 (327 AA) was found only using the AC_RNASeq data but not with the genome data. This strongly suggests some gaps in the assembled reference genome (GenBank assembly accession: GCA_000313135.1). In the case of AP_RNASeq, the performed analysis enabled only for the identification of Mmm1 (569 AA) and Gem1 (711 AA).

**Table 4:**
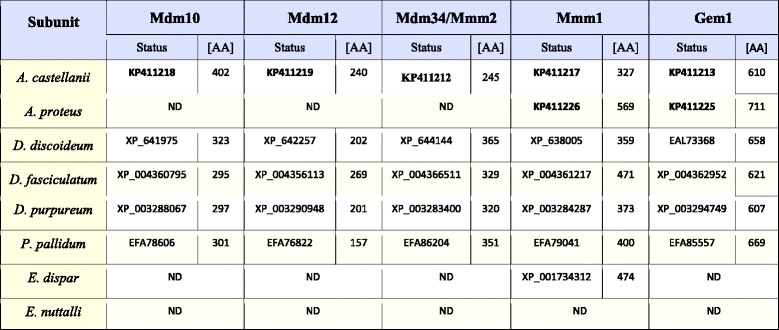
The identified subunits of the studied Amoebozoa ERMES complex

ND indicates proteins not detected in all datasets. Bold numbers denote sequences determined in this study

Available protein datasets and genome data of the studied slime molds confirmed the presence of all canonical subunits of the ERMES complex (Table 4). The following proteins were identified: *D. discoideum* (Mdm10: XP_641975, 323 AA; Mdm12: XP_642257, 202 AA; Mdm34/Mmm2: XP_644144, 365 AA; Mmm1: X_638005, 359 AA; Gem1: EAL73368, 658 AA), *D. fasciculatum* (Mdm10: XP_004360795, 295 AA; Mdm12: XP_004356113, 269 AA; Mdm34/Mmm2: XP_004366511, 329 AA; Mmm1: XP_004361217, 471 AA; Gem1: XP_004362952, 621 AA), *D. purpureum* (Mdm10: XP_003288067, 297 AA; Mdm12: XP_003290948, 201 AA; Mdm34/Mmm2: XP_003283400, 320 AA; Mmm1: XP_003284287, 373 AA; Gem1: XP_003294749, 607 AA), *P. pallidum* (Mdm10: EFA78606, 301 AA; Mdm12: EFA76822, 157 AA; Mmm1: EFA79041, 400 AA; Gem1: EFA85557, 669 AA). Genome data analysis was used to identify Mdm34/Mmm2 of *P. pallidum*. The sequence detected for *P. pallidum* genome turned out to be part of a sequence stored in GenBank under the accession number of EFA86204 (also mentioned by [12]). However, the predicted protein contained 351 AA, whereas the EFA86204 appeared to include 862 AA. Importantly, with the exception of Gem1 of *D. purpureum*, Mdm10 of *D. discoideum* and Mdm12 of *D. purpureum,* all the other subunits of the ERMES complex were previously detected by Flinner et al. (2013) [12], and were identical to those identified by us.

As shown in Table 4, similarity searches performed against protein datasets as well as genomic data for *E. dispar* and *E. nuttalli* indicated only the presence of Mmm1, in the case of *E. dispar* (XP_001734312, 474 AA).

Thus, the obtained data suggested that the organization of the ERMES complex is not the same for the all the amoebozoans studied, as *A. proteus* and *E. dispar* appeared to undergo a strong reduction of the complex subunit content whereas *E. nuttalli* seemed to be depleted of the complex.

### The predicted subunits of the studied complexes display different levels of amino acid sequence conservation

To estimate the amino acid sequences diversity of the predicted subunits between and within the distinguished groups of the Amoebozoa (i.e. amoebas, slime molds and entamoebas) phylogenetic trees were built (Additional file 1: Figure S2). The phylogenetic analysis of the amoeba Tom20 and Tom70 proteins (Additional file 1: Figures S2A and C) did not support the grouping of *A. castellanii* and *A. proteus* together as well as indicated some level of amino acid sequence variability in the case of slime molds, particularly for *D. discoideum* Tom20 and *D. fasciculatum* Tom70. However, in many cases the bootstrap values were rather low causing these groupings not to be reliable. This is most likely the result of a low sequence similarity between the analyzed proteins or imperfect sequence sampling and consequently the very weak phylogenetic signal. However, for Tom40 we observed that amoebas (*A. castellanii and A. proteu*s), slime molds (*D. discoideum, D. purpureum and D. fasciculatum* and *P. pallidum)* and entamoebas (*E. dispar and E. nuttalli*) grouped together although the entamoeba proteins appeared to be distinctly different from the rest of the analyzed Tom40 proteins (Additional file 1: Figure S2B).

In the case of the TOB/SAM complex (Additional file 1: Figures S2D and S2E), both Metaxin and Tob55/Sam50 appeared to be well conserved within amoebas (*A. castellanii and A. proteu*s), and slime molds (*D. discoideum, D. purpureum, D. fasciculatum* and *P. pallidum).* The same applied to Tob55/Sam50 of entamoebas (*E. nuttalli* and *E. dispar*). However, Metaxin of *A. catellanii* and *A. proteus* as well as Tob55/Sam50 of *E. nuttalli* and *E. dispar* seemed to be distinctly different from the rest of the analyzed proteins. Thus, the phylogenetic analysis of these proteins showed on the one hand their difference from the other amoebozoan cognate proteins and on the other hand their distinct similarity within a given group of amoebozoans.

For the predicted subunits of the ERMES complex we observed that Mmm1 of *A. castellanii* and *A. proteus* did not group together (Additional file 1: Figure S2I) although they were located quite close to each other, that suggests some similarities in amino acid sequences. Contrary, Gem1 of *A. castellanii* and *A. proteus* grouped together in the phylogenetic analysis (Additional file 1: Figure S2J). In the case of the slime mold ERMES complex (Additional file 1: Figure S2F-J) grouping of the predicted subunits was obtained between *D. purpureum* and *D. discoideu*m as well as *D. fasciculatum* and *P. pallidum* [35]. Thus, the predicted subunits displayed distinct similarity within groups of slime molds (*D. purpureum, D. discoideum, D. fasciculatum* and *P. pallidum*) and amoebas (*A. castellanii* and *A. proteus*).

To summarize, the obtained results suggested that the predicted subunits of the TOB/SAM and ERMES complexes displayed a high level of conservation within amoebas (*A. castellanii and A. proteus*), slime molds (*D. discoideum, D. purpureum, D. fasciculatum* and *P. pallidum*) and entamoebas (*E. nuttalli and E. dispar*). However, in the case of the TOM complex the predicted subunits differed in amino acid sequences within the group of amoebas and slime molds.

### The intron-exon gene structure of the predicted proteins reflects the diversity of the studied amoebozoans

Transcriptome and genome sequence availability made it possible to define the gene structures for *A. castellanii*, *D. discoideum*, *D. purpureum*, *D. fasciculatum*, *P. pallidum,E. dispar*, and *E. nuttalli* (Additional file 1: Figure S3). The results were summarized in Table 5. Since it has been shown that most of the orthologous genes shared between *D. discoideum* and *E. histolytica* are also shared with plant, animal, and fungal genomes [36, 37], the table also includes model eukaryotic organisms representing fungi, animals and plants.

**Table 5:**
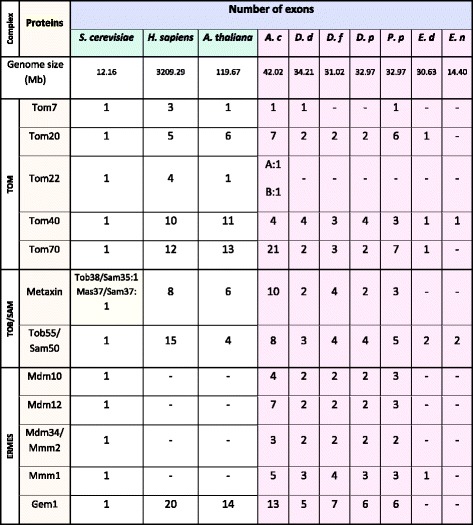
The exon numbers in genes encoding the identified subunits for the studied complexes of Amoebozoa and representatives of fungi, animals and plants

*A. castellanii* (*A. c*), *A. proteus (A.p), D. discoideum* (*D.d)*, *D. fasciculatum* (*D. f)*, *D. purpureum* (*D. p*), *P. pallidum* (*P. p*), *E. dispar* (*E. d) and E. nuttalli* (*E. n*). Minus sign denotes the lack of data enabling determination of the exon numbers

As shown in Table 5, the genes coded for Tom7 and identified for *A. castellanii, D. discoideum,* and *P. pallidum* contained no introns (Additional file 1: Figure S3A). The same was observed for the gene encoding Tom7 of *A. thaliana* and *S. cerevisiae,* whereas the gene of *H. sapiens* consisted of three exons. For genes encoding Tom20 of the studied amoebozoans various numbers of exons were detected (Additional file 1: Figure S3B); namely, seven for *A. castellanii*, two for *D. discoideum, D. fasculatum* and *D. purpureum,* six for *P. pallidum* and one for *E. dispar.* In the case of *S. cerevisiae,* the gene contained one exon, whereas for *H. sapiens* and *A. thaliana* the number of exons was comparable, (five and six, respectively). Two genes encoding Tom22 were detected only for *A. castellanii* and they both consisted of a single exon as in the case of *S. cerevisiae* and *A. thaliana,* whereas the gene of *H. sapiens* contained four exons. The gene coding for Tom40 (Additional file 1: Figure S3C) of *A. castellanii, D. discoideum,* and *D. purpureum,* consisted of four exons. In the case of *D. fasciculatum* and *P. pallidum,* the gene contained three exons but in the case of *E. dispar* and *E. nuttalli,* consisted of only one exon. The lack of introns was also observed for the *S. cerevisiae* gene, whereas *H. sapiens* and *A. thaliana* genes contained ten and eleven exons, respectively. The Tom70 gene exhibited large variations in length and numbers of exons (Additional file 1: Figure S3D). The gene of *A. castellanii* displayed the presence of twenty-one exons, whereas the gene of *D. discoideum*, *D. purpureum* and *D. fasciculatum* contained two and three exons, respectively. The *P. pallidum* gene contained seven exons, whereas the gene of *E. dispar* was without introns, similar to the gene of *S. cerevisiae*. Interestingly, just as for *A. castellanii*, multiple exons were detected for *A. thaliana* and *H. sapiens* Tom70 (thirteen and twelve, respectively).

Multiple exons were also predicted for the *A. castellanii* Metaxin gene (Additional file 1: Figure S3E) and the Tob55/Sam50 gene (Additional file 1: Figure S3F). The genes consisted of ten and eight exons, respectively (Table 5). For *D. discoideum* and *D. purpureum,* the gene encoding Metaxin contained two exons, whereas the number of predicted exons for the *D. fasciculatum* and *P. pallidum* genes was four and three, respectively. As *S. cerevisiae* do not possess the Metaxin gene, the number of exons was determined only for the *H. sapiens* and *A. thaliana* genes containing eight and six exons, respectively. The Tob55/Sam50 encoding gene of *D. discoideum* contained three exons but the gene of *D. purpureum* and *D. fasciculatum* contained four exons, and the gene of *P. palldium* five exons. For *E. dispar* and *E. nuttalli* two exons were detected in the gene (Additional file 1: Figure S3F) whereas the gene of *H. sapiens* and *A. thaliana* consisted of fifteen and four exons, respectively.

As shown in Table 5, the canonical subunits of the ERMES complex were analyzed for *S. cerevisiae* and the studied amoebozoans with the exception of Gem1, which has been proven to be the only subunit of the complex present in nearly all multicellular eukaryotes [12]. Interestingly, all the analyzed genes for *S. cerevisiae* consisted of only one exon. The gene encoding Mdm10 contained four exons for *A. castellanii* and three exons for *P. pallidum*, whereas for *D. discoideum*, *D. fasciculatum* and *D. purpureum* the gene contained only two exons (Additional file 1: Figure S3G). The gene encoding Mdm12 of *A. castellanii* consisted of seven exons, but for *P. pallidum* the number of exons was three, whereas the *D. discoideum*, *D. fasciculatum* and *D. purpureum* cognate genes contained two exons (Additional file 1: Figure S3H). The gene for Mdm34/Mmm2 protein of *A. castellanii* contained three exons, whereas the *D. discoideum*, *D. fasciculatum*, *D. purpureum* and *P. pallidum* genes harboured two exons (Additional file 1: Figure S3I). The predicted exon-intron structure of Mmm1 gene indicated higher number of exons for *A. castellanii* and *D. fasciculatum* (five and four respectively) when compared with the predicted number of three exons for the rest of the studied slime molds (Additional file 1: Figure S3J). Interestingly, Mmm1 encoding gene of *E. dispar* contained no introns. The gene encding Gem1 contained thirteen exons in the case of *A. castellanii*, seven for *D. fasciculatum,* six exons in the case of *D. purpureum* and *P. palldium,* and five for *D. discoideum* (Additional file 1: Figure S3K)*.* Multiple exons were detected for the *A. thaliana* and *H. sapiens* Gem1 gene, namely fourteen and twenty, respectively.

To summarize, the predicted number of exons for genes encoding subunits of the TOM, TOB/SAM and ERMES complexes appeared to be consistent within group of slime molds (*D. purpureum, D. discoideum, D. fasciculatum* and *P. pallidum*) and entamoebas (*E. dispar* and *E. nuttalli*). Moreover, the number of predicted exons supported the position of amoebozoans between fungi and multicellular eukaryotes observed in the phylogenetic trees [35]. Interestingly, the number of exons predicted for entamoeba genes was most similar to that of *S. cerevisiae,* whereas *A. castellanii* seemed to be more similar to plants and animals.

### Phylogenetic position of the predicted subunits of the TOM, TOB/SAM and ERMES complexes

Since Amoebozoa is regarded to be the most closely related to Opisthokonta, which includes Choanozoa, animals and fungi, we would expect the predicted subunits of the TOM, TOB/SAM and ERMES complexes to be most similar to the cognate proteins of fungi, choanozoa and animals. Therefore, we collected proteins of other organisms, which displayed highest sequence similarity to the predicted amoebozoan subunits of the TOM, TOB/SAM and ERMES complexes (Tables 6, 7, and 8, respectively). In regards to the TOM complex, highest similarity to Opisthokonta proteins was observed for predicted Tom7, Tom22, and Tom70 although the cognate proteins represented different Opisthokonta lineages. However, Tom22 predicted for *A. castellanii* showed only week similarity to known Tom22 sequences as e-value obtained for the best match (XP_011141090) was 1.8 (not shown in Table 6). Unexpectedly, the predicted Tom20 displayed highest similarity to the Archaeplastida (plant) proteins. The same was true for Tom40 of *A. castellanii* and *D. discoideum*. Moreover, Tom40 of *E. dispar* and *E. nuttalli* was most similar to the Excavata protein. However, the data collected for the TOB/SAM and ERMES complexes indicated that the predicted subunits showed highest similarity to the cognate proteins of Opisthokonta usually representing the same Opisthokonta lineage. The data appeared to confirm results of phylogenetic analysis of the predicted subunits of the studied complexes (Additional file 1: Figure S2), indicating the highest level of amino acid sequence diversity for Tom proteins.

**Table 6:**
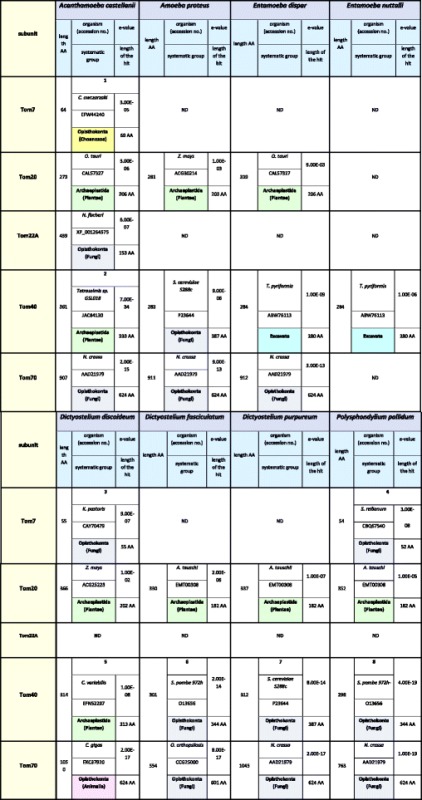
The highest similarity reference for sequences of the identified TOM complex proteins to the applied reference sequences

ND denotes not detected. Numbers indicate the highest similarities in the Amoebozoa group with: 1: KM655837 *A. castellanii*; 2: ADZ24223 *A. castellanii*; 3: XP_639342 *D. discoideum*, EFA78398 *P. pallidum*; 4: EFA78398 *P. pallidum*, XP_639342 *D. discoideum*; 5: XP_642798 *D. discoideum*, EFA80126 *P. pallidum*; 6: XP_004352318 *D. fasciculatum*, XP_642798 *D. discoideum*; 7: XP_642798 *D. discoideum*; 8: EFA80126 *P. pallidum*, XP_642798 *D. discoideum*

**Table 7:**
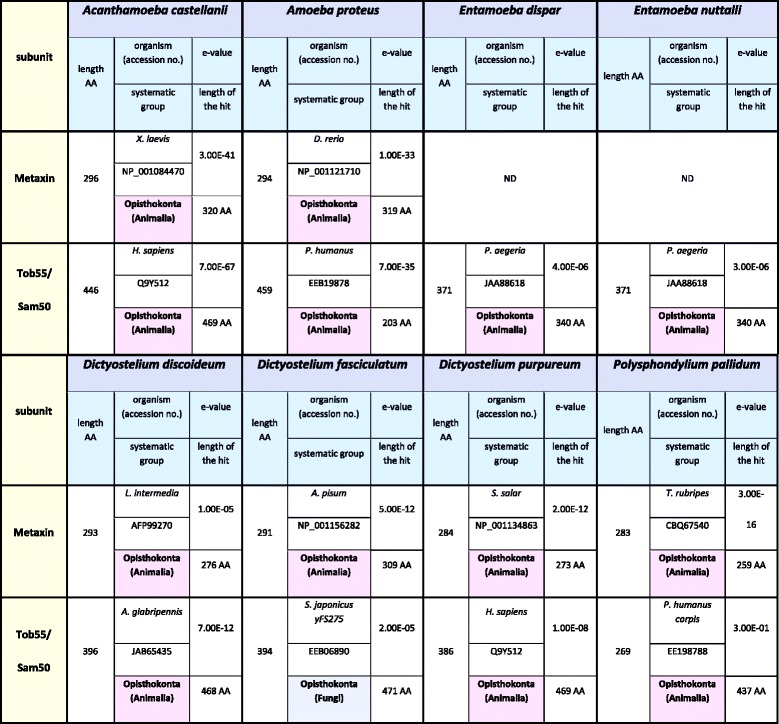
The highest similarity reference for sequences of the identified TOB/SAM complex proteins to the applied reference sequences

ND denotes not detected

**Table 8:**
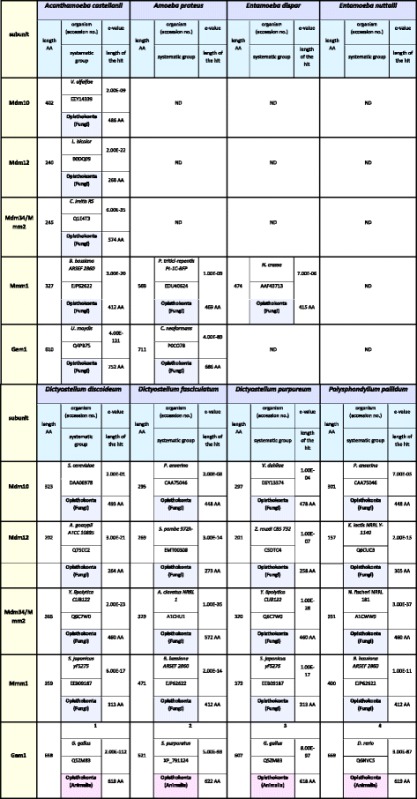
The highest similarity reference for sequences of the identified ERMES complex proteins to the applied reference sequences

ND denotes not detected. Numbers indicate the highest similarities in the Amoebozoa group with: 1: XP_647338 *D. discoideum*, F1A505 *D. purpureum*, EGG25101 *D. fasciculatum*, EFA85557 *P. pallidum*; 2: EGG25101 *D. fasciculatum*, EFA85557 *P. pallidum*, XP_647338 *D. discoideum*, F1A505 *D. purpureum*; 3: F1A505 *D. purpureum*, XP_647338 *D. discoideum*, EGG25101 *D. fasciculatum*, EFA85557 *P. pallidum*; 4: EFA85557 *P. pallidum*, EGG25101 *D. fasciculatum*, XP_647338 *D. discoideum*, F1A505 *D. purpureum*

## Discussion

The TOM, TOB/SAM and ERMES complexes have been intensively studied in *Saccharomyces cerevisiae* applied as a model system to explain mechanisms of their contribution to mitochondria biogenesis (e.g. [14]). However, there is still not enough data to address the evolutionary aspects of the import machinery functioning. Differences in the subunit organization of the complexes have been observed for representatives of different phylogenetic lineages but for representatives of the same clade (supergroup) grouped into distinct subclades and divisions the issue is not so well known. Accordingly, the Amoebozoa represent one of the systematic groups that are poorly sampled for mitochondrial protein import complexes. Importantly, the Amoebozoa encompass taxa of both biomedical and evolutionary importance, yet its genomic, transcriptomic diversity remains largely unsampled.

Bioinformatic analyses demonstrated the presence of Tom40, Tom60, a novel lineage-specific receptor protein, and Tob55/Sam50 in *Entamoeba* species [17, 34, 35, 38]. However, it should be noted that most entamoebas are pathogentic species which posses mitosomes instead of mitochondria with dramatically reduced import machineries [25, 39]. This fact notwithstanding, *Entamoeba dispar* and *Entamoeba nuttalli* are not pathogenic and are now recognized as separate species from pathogenic *Entamoeba histolytica* and *Entamoeba invadens* [40]. For *Acanthamoeba castellanii,* only the presence of Tom40, Tom7, Tob55/Sam50 and Tom70 has been reported so far [34, 35]. In the case of the slime mold TOM and TOB/SAM complexes, available data indicate the presence of Tom40, Tom22, and Tom7 as well as Tob55/Sam50 for *Dictyostelium discoideum* [17, 33, 35, 41], Tom7, Tom40 and Tob55/Sam50 for *Pollysphondylium pallidum* [34, 35], and Tom40 and Tob55/Sam50 for *Dictyostelium purpureum* [35]. On the other hand, the presence of the ERMES complex subunits, i.e. Gem1, Mdm10, Mdm12, Mdm34/Mmm2 and Mmm1 (with some exceptions) has been shown for *Dictyostelium discoideum, Dictyostelium fasciculatum, Dictyostelium purpureum* and *Pollysphondylium pallidum* [12]. Therefore, we performed analysis of genome and transcriptome sequences available for different amoebozoans as a verification of the attainable data to build a more coherent picture of the Amoebozoa protein import complexes in the mitochondrial outer membrane. The studied organisms represent different subclades and subdivisions of the Amoebozoa [30]. *A. castellanii* and *A. proteus* represent Lobosa (Discosea and Tubulinea, respectively), whereas the remainder belong to Conosa: *D. discoideum, D. purpureum, D. fasciculatum* and *P. pallidum* are classified as Mycetozoa and *E. dispar* and *E. nuttalli* as Archamoebea.

The obtained results indicate differences in subunit organization of the studied complexes even in the case of representatives of the same subclade and subdivision (Tables 2, 3, and 4). The TOM complex of the studied amoebozoans appeared to contain one to five subunits. Consequently, the only subunit identified for all TOM complexes studied is Tom40. The latter is the only identified subunit of the *E. nuttalli* TOM complex, although the *E. dispar* TOM complex is shown to contain Tom20, Tom40 and Tom70. The slime mold TOM complexes are predicted to contain three to four subunits. The obtained data indicate the presence of Tom20, Tom40 and Tom70 in all complexes. The absence of Tom7 is observed for *D. purpureum* and *D. fasciculatum,* whereas the protein is present in the *D. discoideum* and *P. pallidum* TOM complexes*.* Accordingly, the TOM complex of *A. proteus* appears to contain three subunits (Tom20, Tom40 and Tom70), while in the case of *A. castellanii* the available data indicate the presence of five subunits (Tom7, Tom20, Tom22, Tom40 and Tom70) (Table 2). Less diversity is observed for the ERMES complex. All the postulated subunits of the complex, i.e. Mdm10, Mdm12, Mdm34/Mmm2, Gem1 and Mmm1, the latter being ER membrane protein (e.g. [12]), are found for the slime molds. The same applies to the *A. castellanii* ERMES complex. However, the *A. proteus* ERMES complex does not contain Mdm10, Mdm12, Mdm34/Mmm2. Moreover, the complex appears not to be present in *E. nuttalli,* and only the presence of Mmm1 is observed for *E. dispar* (Table 4). Thus, the ERMES complex does not appear to be present in all representatives of the Amoebozoa [12]. On the other hand, the predicted organization of the TOB/SAM complex appears to be identical for all studied amoebozoans with the exception of the entamoeba complex, which lacks Metaxin (Table 3). However, it should be emphasized that sequences of the involved proteins could have changed significantly in the course of evolution resulting in proteins difficult to identify *in silico* and/or some of the proteins could have been replaced by components not yet determined [42].

Interestingly, the number of the TOM complex identified subunits is highest in the case of *A. castellanii*. Accordingly, the *A. castellanii* TOM complex includes Tom22, not present in the other amoebozoans. The presence of the protein has been suggested for *D. discoideum* (e.g. [17, 33, 38, 41]), but has not been confirmed till now (P. Dolezal, personal communication). The putative *A. castellanii* Tom22 which is identical to the sequence deposited in GenBank as XP_004353494 contains 413 amino acids. Importantly, canonical Tom22 of *S. cerevisiae* (GenBank gi: 285814523) has a molecular weight of 16.8 kDa and consists of 152 amino acids. Therefore, out of two Tom22 candidates detected in this study, the shorter form (Tom22 B) resembles the canonical one better than the longer form (Tom22 A). However, both forms display high sequence similarity in the shared region and without further experiments it is impossible to decide which of the forms is active member of the TOM complex.

On the other hand, we observed differences between *A. castellanii* Tob55/Sam50, Metaxin and Gem1 proteins predicted with the aid of AC_RNASeq analysis and available protein datasets for *A. castellanii* [43]. As shown in Supplementary file (Additional file 1: Figure S1), all the predicted proteins display a high level of amino acid sequence identity, although simultaneously differ by the presence of unique blocks of amino acid sequences. Thus, the amino acid sequences of Metaxin, Tob55/Sam50 and Gem1 are longer than those deposited in GenBank (XP_004337900, XP_004341043 and XP_004356731, respectively). The discrepancies may be due to sequencing and/or assembly errors on both sites.

The way of clustering of the studied proteins in phylogenetic trees (Additional file 1: Figure S2) suggests that the analyzed Tom proteins differ in amino acid sequences within the group of amoebas (*A. castellanii and A. proteus*) and slime molds (*D. discoideum, D. purpureum, D. fasciculatum and P. pallidum*). However, the predicted subunits of the TOB/SAM complex display a high level of conservation within amoebas (*A. castellanii and A. proteus*), slime molds (*D. discoideum, D. purpureum, D. fasciculatum* and *P. pallidum*) and entamoebas (*E. nuttalli and E. dispar*). The same applies to subunits of the ERMES complex within the groups of slime molds (*D. purpureum, D. discoideum, D. fasciculatum* and *P. pallidum*) and amoebas (*A. castellanii* and *A. proteus*). Interestingly, the predicted subunits of the TOB/SAM and ERMES complexes display highest similarity to the cognate proteins of Opisthokonta (Tables 7 and 8). This is in agreement with the proposed close relationship between the Opisthokonta and the Amoebozoa [26–28]. However, in the case of some Tom proteins, a similarity to plant and Excavata proteins is also observed (Table 6). Altogether, the data confirm the diversity of Tom proteins and higher similarity of subunits of the TOB/SAM and ERMES complexes. This constitutes an interesting issue from the evolutionary perspective as it addresses the problem of mitochondrial protein import machinery variability within currently defined supergroups of eukaryotes. The Amoebozoa comprises a wide variety of amoeboid and flagellate organisms with single cells of various sizes that have adopted different lifestyles and live in different environments. Thus, taking into account the TOM complex localization and function, it can be speculated that the complex contributes importantly to adaptation evolution and its variability results from the natural selection. The variations observed between representatives of different supergroups are regarded to mirror the early diversification of eukaryotes [44]. By analogy, in the case of a given supergroup, it may provide important information concerning its branches. On the other hand, it is suggested that ERMES subunits occurred concomitantly in unikonts, including Opisthokonta and Amoebozoa [45], and then were lost in animals and plants, but are still present in representatives of Amoebozoa [12]. Our result indicate that the reduction may also apply to amoebozoans although it is difficult to explain at least for *A. proteus* as in the case of entamoebas it may result from conversion of their mitochondria to mitosomes.

As mentioned above, the amino acid sequence of the predicted proteins (Tables 2, 3, and 4) differ between and within the studied groups of amoebozoans, i.e. amoebas, slime molds and entamoebas (Additional file 1: Figure S2). Therefore, we performed an analysis of intron-exon structure of the putative genes and compared the obtained results to the data of fungi, plants and animals known to share orthologous gene families with the Amoebozoa representatives [37]. It should be noted that the canonical subunits of the ERMES complex, with the exception of the Gem1 protein, are lost in plants and animals [12]. As shown in Additional file 1: Figure S3 and summarized in Table 5, the analyzed genes display high diversity in regards to the predicted number of exons and consequent number of introns. The numbers seem to be distinctive for a given group of the studied amoebozoans, rather than for a given gene. Moreover, the number of predicted exons seems to support the phylogenetic positioning of the amoebozoans between fungi and multicellular eukaryotes represented by *A. thaliana* (plants) and *H. sapiens* (animals). The determined number of exons for entamoeba genes is most similar to that which is known for *S. cerevisiae,* whereas *A. castellanii* appears to be more similar in the respect to plants and animals. Accordingly, the existence of multiple exon structures in the case of *A. castellanii,* constitutes a very interesting issue from the point of view of the species evolution. Because of its life style *A. castellanii* can be defined as a phagotroph. The group of organisms encounters a rich and diverse supply of foreign DNA that provides opportunity for lateral gene transfer [46]. Indeed, *A. castellanii* genes have an average of 6.2 introns per gene, among the highest known in eukaryotes although it has been shown that these events have been very rare for *A. castellanii* [43]. On the other hand, it is known that different species have evolved considerably different intron–exon structures and these, in turn, are correlated with the evolution of genomes and are constrained by functional properties of intron splicing processes [47]. These imply different regulation of protein expression at the level of splicing supporting an adaptation to a given life style. Correspondingly, *A. castellanii* is the only parasite among the studied amoebozoans and differ from the other studied amoebozoans by multiple exon structure. Nevertheless, the obtained results seems to support the division of the amoebozoans into subclades of Conosa (and further into Archamoebea and Mycetozoa, represented by entamoebas and slime molds, respectively) and Lobosa (represented by *A. castellanii* (e.g. [30]).

Interestingly, the obtained results indicate reduced organization of the TOM, TOB/SAM and ERMES complexes in the case of *E. dispar* and *E. nuttalli*, particularly pronounced for *E. nuttalli* (Tables 2, 3, and 4). It has been shown that extreme reduction of the mitochondrial protein import apparatus is characteristic for mitosomes, being metabolically specialized forms of mitochondria which evolved as a response to anaerobic and partly parasitic lifestyles in diverse eukaryotic lineages (e.g. [41]). As a matter of fact, the presence of mitosomes has been proven for *E. invadens* and distantly related *E. histolytica*. Moreover, the presence of mitosomes has been suggested for all *Entamoeba* spp. [41, 48]. Accordingly, the genomes of *E. dispar* and *E. nuttalli* contain genes for the proteins found in mitosomes of *E. histolytica* and *E. invadens*, and presumably, all of these species have mitosomes, although convincing data are not yet available (Graham Clark, personal communication). The organization of the TOM and TOB/SAM complexes of *E. nuttalli* strongly resembles those described for *E. histolytica* [34, 41]. In contrast, proteins predicted for *E. dispar* indicate a less advanced reduction of the complexes that may reflect a less advanced transition between mitochondria and mitosomes.

## Conclusions

In this study, we presented comprehensive bioinformatic analyses of the TOM, TOB/SAM and ERMES complexes of the Amoebozoa representatives based on searching of available genome and transcriptome data. The obtained results clearly demonstrate structural variability/diversity of these complexes in the Amoebozoa lineage and the reduction of their complexity as compared with the same complexes of model organisms. The results contribute to the evolutionary discussion on mitochondrial protein import mechanism, especially concerning the elimination of proteins involved in crucial stages of the process and the possibility of the protein replacement by new, functionally equivalent ones.

## Methods

### The studied amoebozoans

The organisms studied were as follows: *Acanthamoeba castellanii* and *Amoeba proteus* (Lobosa*;* Discosea and Tubulinea, respectively), *Dictyostelium discoideum, Dictyostelium purpureum, Dictyostelium fasciculatum* and *Polysphondylium pallidum* (Conosa; Mycetozoa), as well as *Entamoeba dispar* and *Entamoeba nuttalli* (Conosa; Archamoebea). Table 1 summarizes the availability of data concerning sequences of their genome and transcriptomes.

### *Acanthamoeba castellanii* and *Amoeba proteus* cell cultures and isolation of total RNA

Cells of *Acanthamoeba castellanii* (strain Neff) were cultured at 28 °C, in an axenic environment in the standard medium described by Neff [49], with some modifications: 1.5 % proteoso-pepton, 0.15 % yeast extract, 30 mM MgCl_2_, 30 mM FeSO_4_, 27 mM CaCl_2_, 1.5 % glucose, 2.5 mg/l vitamin B12, 1 mg/l vitamin B1, 0.2 mg/l vitamin H. Cells in the trophozite stage were collected in the intermediary phase after 48 h and were frozen in liquid nitrogen and homogenized in TRIzol reagent (Invitrogen). Total RNA was isolated according to the manufacturer’s instructions (Invitrogen). DNaseI was added to eliminate remaining genomic DNA. The absence of DNA was confirmed by PCR and agarose gel electrophoresis.

*Amoeba proteus* cells were cultured in KCM medium (7 mg/l KCl, 8 mg/l CaCl_2_, and 8 mg/l MgSO_4_ x 7H_2_O) at 25 °C and fed with *Tetrahymena pyriformis* twice a week. To avoid contamination of the *T. pyriformis*, the medium was changed daily. *A. proteus* cells were starved seven days before total RNA isolation performed as described above for *A. castellanii.*

### *A. castellanii and A. proteus* cDNA preparation, sequencing and assembly of transcriptomes

cDNA was prepared using a mRNA-Seq Sample preparation Kit (Illumina) and according to the manufacturer’s instructions. Sequencing of the cDNA, i.e. mRNA-Seq of *A. castellanii* (AC_RNASeq, Buczek et. al, unpublished data) and *A. proteus* (AP_RNASeq, Sonobe et al., unpublished data) were performed on the HiSeq 2000 platform (Illumina) with 36 bp single end reads and 101 bp paired ends, respectively. The obtained raw reads were subjected to quality control analysis using standard tools provided by Illumina. Over 219 million of *A. castealanii* reads and 129 million of *A. proteus* reads were obtained. In the case of *A. castealanii* we were able to map raw reads to the existing genome with 65 % rate and 0.5 % mismatch rate. After removal of poor quality sequences (about four per cent), short reads were assembled using Trinity RNA-Seq [50] with the following parameters: −-SS_lib_type FR for *A. proteus* (AP_TRANS), F for *A. castellanii* (AC_TRANS) and for the both organisms --min_contig_length 300.

### Identification/prediction of proteins

To find the best annotated protein sequences for subunits of the TOM, TOB/SAM and ERMES complexes, keyword searches against the NCBI (http://www.ncbi.nlm.nih.gov/) and Pfam (http://pfam.sanger.ac.uk/) databases were performed. First, sets of well-known sequences from different species representing various eukaryotic lineages (Additional file 1: Table S1) were used as queries in tBLASTn searches [51] against the transcriptome of *A. castellanii* with variable e-values (from 10^−3^ to 1). For proteins which were not identified by tBLASTn, a HMMER search based on Hidden Markov Models was performed [52]. In the case a reference sequence showed a significant similarity to several contigs, a sequence with the longest potential CDS was selected. Such a CDS was further confirmed by a reciprocal BLAST against NCBI nr database and/or pfam domain search. To translate transcripts to the protein sequences the ExPASY server was used and for each sequence different reading frames were checked to find the longest and more likely sequences of TOM, TOB/SAM and ERMES subunits [53]. The amino acid sequences of putative proteins were subjected to a BLASTp [51] search in order to compare the sequences with available protein datasets of *A. castellanii* [43]. To find previously un-annotated proteins, a tBLASTn search against the available genome of *A. castellanii* was performed.

Subsequently, proteins identified for *A. castelanii* were used in a tBLASTn search against sequenced transcriptomes of *A. proteus* and for a BLASTp search against the protein datasets of *Dictyostelium purpureum, Dictyostelium discoideum, Dictyostelium fasciculatum*, *Pollysphondylium pallidum*, *Entamoeba dispar* and *Entamoeba nuttalli*. For proteins, which were not identified by the analysis, tBLASTn algorithm was used against the available genomes of *D. discoideum*, *D. purpureum*, *D. fasciculatum*, *P. pallidum*, *E. dispar and E. nuttalli*. Finally, reference sequences from various eukaryotic lineages (Additional file 1: Table S1) were used to identify proteins not found by the previously applied methods.

### The intron – exon gene structure analysis

The intron/exon gene structures were determined by SPLIGN [54]. The gene sequences were aligned by the Multiple Sequences Alignment (MSA) - program MUSCLE 3.8.31 [55]. The predicted exons were mapped to protein sequences and then AlignExIn, an in-house tool was implemented (www.bioinformatics.uni-muenster.de/tools/alignexin/).

### Phylogenetic inference

To position the relationships of the analyzed amoebozoan proteins, unrooted phylogenetic trees were calculated using RAxML 7.0.4 with default parameters and 1000 bootstraps [56]. To visualize and edit the obtained phylogenetic trees, FigTree version 1.4.2 (http://tree.bio.ed.ac.uk/software/figtree/) was used.

### Signal peptide analysis

Signal peptides were predicted using the SignalP 4.1 Server at http://www.cbs.dtu.dk/services/SignalP/ [57].

### Neutrality test

The numbers of synonymous and nonsynonymous differences between sequences were estimated using the Nei-Gojobori method [58] as implemented in MEGA5.2 software [59]. The same software was likewise used for the neutrality test.

## Additional file

Additional file 1: Table S1. Contains a table with the information on the reference sequences used as queries in tBLASTn searches against the transcriptome of *A. castelanii*, alignments of the identified subunits of theTOM, TOB/SAM and ERMES complexes in and their counterparts deposited in the GenBank and displaying differences in amino acid sequences, phylogenetic tress of the identified subunits, and graphical representation of the intron – exon gene structure for the identified subunits. (PDF 2670 kb)

## References

[1] Dolezal P, Likic V, Tachezy J, Lithgow T (2006). Evolution of the molecular machines for protein import into mitochondria. Science.

[2] Embley TM, Martin W (2006). Eukaryotic evolution, changes and challenges. Nature.

[3] Schmidt O, Pfanner N, Meisinger C (2010). Mitochondrial protein import: from proteomics to functional mechanisms. Nat Rev Mol Cell Biol.

[4] Neupert W, Herrmann JM (2007). Translocation of proteins into mitochondria. Annu Rev Biochem.

[5] Chacinska A, Koehler CM, Milenkovic D, Lithgow T, Pfanner N (2009). Importing mitochondrial proteins: machineries and mechanisms. Cell.

[6] Endo T, Yamano K (2010). Transport of proteins across or into the mitochondrial outer membrane. Biochim Biophys Acta.

[7] Dukanovic J, Rapaport D (2011). Multiple pathways in the integration of proteins into the mitochondrial outer membrane. Biochim Biophys Acta.

[8] Becker T, Bottinger L, Pfanner N (2012). Mitochondrial protein import: from transport pathways to an integrated network. Trends Biochem Sci.

[9] Neupert W (2015). A perspective on transport of proteins into mitochondria: a myriad of open questions. J Mol Biol.

[10] Kornmann B, Walter P (2010). ERMES-mediated ER-mitochondria contacts: molecular hubs for the regulation of mitochondrial biology. J Cell Sci.

[11] Michel AH, Kornmann B (2012). The ERMES complex and ER-mitochondria connections. Biochem Soc Trans.

[12] Flinner N, Ellenrieder L, Stiller SB, Becker T, Schleiff E, Mirus O (2013). Mdm10 is an ancient eukaryotic porin co-occurring with the ERMES complex. Biochim Biophys Acta.

[13] Wideman JG, Lackey SW, Srayko MA, Norton KA, Nargang FE (2013). Analysis of mutations in Neurospora crassa ERMES components reveals specific functions related to beta-barrel protein assembly and maintenance of mitochondrial morphology. PLoS One.

[14] Sokol AM, Sztolsztener ME, Wasilewski M, Heinz E, Chacinska A (2014). Mitochondrial protein translocases for survival and wellbeing. FEBS Lett.

[15] Kornmann B, Currie E, Collins SR, Schuldiner M, Nunnari J, Weissman JS (2009). An ER-mitochondria tethering complex revealed by a synthetic biology screen. Science.

[16] Stroud DA, Oeljeklaus S, Wiese S, Bohnert M, Lewandrowski U, Sickmann A (2011). Composition and topology of the endoplasmic reticulum-mitochondria encounter structure. J Mol Biol.

[17] Lithgow T, Schneider A (2010). Evolution of macromolecular import pathways in mitochondria, hydrogenosomes and mitosomes. Philos Trans R Soc Lond Ser B Biol Sci.

[18] Hewitt V, Alcock F, Lithgow T (2011). Minor modifications and major adaptations: the evolution of molecular machines driving mitochondrial protein import. Biochim Biophys Acta.

[19] Liu Z, Li X, Zhao P, Gui J, Zheng W, Zhang Y (2011). Tracing the evolution of the mitochondrial protein import machinery. Comput Biol Chem.

[20] Pusnik M, Mani J, Schmidt O, Niemann M, Oeljeklaus S, Schnarwiler F (2012). An essential novel component of the noncanonical mitochondrial outer membrane protein import system of trypanosomatids. Mol Biol Cell.

[21] Murcha MW, Wang Y, Narsai R, Whelan J (2014). The plant mitochondrial protein import apparatus - the differences make it interesting. Biochim Biophys Acta.

[22] Gentle I, Gabriel K, Beech P, Waller R, Lithgow T (2004). The Omp85 family of proteins is essential for outer membrane biogenesis in mitochondria and bacteria. J Cell Biol.

[23] Zeth K (2010). Structure and evolution of mitochondrial outer membrane proteins of beta-barrel topology. Biochim Biophys Acta.

[24] Bay DC, Hafez M, Young MJ, Court DA (2012). Phylogenetic and coevolutionary analysis of the beta-barrel protein family comprised of mitochondrial porin (VDAC) and Tom40. Biochim Biophys Acta.

[25] Heinz E, Lithgow T (2013). Back to basics: a revealing secondary reduction of the mitochondrial protein import pathway in diverse intracellular parasites. Biochim Biophys Acta.

[26] Keeling PJ, Burger G, Durnford DG, Lang BF, Lee RW, Pearlman RE (2005). The tree of eukaryotes. Trends Ecol Evol.

[27] Dacks JB, Walker G, Field MC (2008). Implications of the new eukaryotic systematics for parasitologists. Parasitol Int.

[28] Adl SM, Simpson AG, Lane CE, Lukes J, Bass D, Bowser SS (2012). The revised classification of eukaryotes. J Eukaryot Microbiol.

[29] Smirnov AV, Chao E, Nassonova ES, Cavalier-Smith T (2011). A revised classification of naked lobose amoebae (Amoebozoa: lobosa). Protist.

[30] Fiz-Palacios O, Romeralo M, Ahmadzadeh A, Weststrand S, Ahlberg PE, Baldauf S (2013). Did terrestrial diversification of amoebas (amoebozoa) occur in synchrony with land plants?. PLoS One.

[31] Emanuelsson O, Brunak S, von Heijne G, Nielsen H (2007). Locating proteins in the cell using TargetP, SignalP and related tools. Nat Protoc.

[32] Rodriguez-Cousino N, Nargang FE, Baardman R, Neupert W, Lill R, Court DA (1998). An import signal in the cytosolic domain of the Neurospora mitochondrial outer membrane protein TOM22. J. Biol. Chem..

[33] Macasev D, Whelan J, Newbigin E, Silva-Filho MC, Mulhern TD, Lithgow T (2004). Tom22', an 8-kDa trans-site receptor in plants and protozoans, is a conserved feature of the TOM complex that appeared early in the evolution of eukaryotes. Mol Biol Evol.

[34] Makiuchi T, Mi-ichi F, Nakada-Tsukui K, Nozaki T (2013). Novel TPR-containing subunit of TOM complex functions as cytosolic receptor for Entamoeba mitosomal transport. Sci. Rep..

[35] Wojtkowska M, Jakalski M, Pienkowska JR, Stobienia O, Karachitos A, Przytycka TM (2012). Phylogenetic analysis of mitochondrial outer membrane beta-barrel channels. Genome Biol. Evol..

[36] Glockner G, Noegel AA (2013). Comparative genomics in the Amoebozoa clade. Biol Rev Camb Philos Soc.

[37] Song J, Xu Q, Olsen R, Loomis WF, Shaulsky G, Kuspa A (2005). Comparing the Dictyostelium and Entamoeba genomes reveals an ancient split in the Conosa lineage. PLoS Comput Biol.

[38] Likic VA, Dolezal P, Celik N, Dagley M, Lithgow T (2010). Using hidden markov models to discover new protein transport machines. Methods Mol Biol.

[39] Mi-ichi F, Abu Yousuf M, Nakada-Tsukui K, Nozaki T (2009). Mitosomes in Entamoeba histolytica contain a sulfate activation pathway. Proc Natl Acad Sci U S A.

[40] Ackers JP (2002). The diagnostic implications of the separation of Entamoeba histolytica and Entamoeba dispar. J Biosci (Suppl 3).

[41] Dolezal P, Dagley MJ, Kono M, Wolynec P, Likic VA, Foo JH (2010). The essentials of protein import in the degenerate mitochondrion of Entamoeba histolytica. PLoS Pathog.

[42] Eckers E, Cyrklaff M, Simpson L, Deponte M (2012). Mitochondrial protein import pathways are functionally conserved among eukaryotes despite compositional diversity of the import machineries. Biol Chem.

[43] Clarke M, Lohan AJ, Liu B, Lagkouvardos I, Roy S, Zafar N (2013). Genome of Acanthamoeba castellanii highlights extensive lateral gene transfer and early evolution of tyrosine kinase signaling. Genome Biol.

[44] Mani J, Meisinger C, Schneider A. Peeping at TOMs-Diverse Entry Gates to Mitochondria Provide Insights into the Evolution of Eukaryotes. Mol Biol Evol. 2016;33(2):337-51. Epub 2015. 10.1093/molbev/msv219 26474847

[45] Cavalier-Smith T (2002). The phagotrophic origin of eukaryotes and phylogenetic classification of Protozoa. Int J Syst Evol Microbiol.

[46] Keeling PJ, Palmer JD (2008). Horizontal gene transfer in eukaryotic evolution. Nat Rev Genet.

[47] Deutsch M, Long M (1999). Intron-exon structures of eukaryotic model organisms. Nucleic Acids Res.

[48] Siegesmund MA, Hehl AB, van der Giezen M (2011). Mitosomes in trophozoites and cysts of the reptilian parasite Entamoeba invadens. Eukaryotic cell.

[49] Neff RJ (1957). Purification, axenic cultivation, and description of a soil amoeba, Acanthamoeba sp. J Protozool.

[50] Grabherr MG, Haas BJ, Yassour M, Levin JZ, Thompson DA, Amit I (2011). Full-length transcriptome assembly from RNA-Seq data without a reference genome. Nat Biotechnol.

[51] Altschul SF, Gish W, Miller W, Myers EW, Lipman DJ (1990). Basic local alignment search tool. J Mol Biol.

[52] Eddy SR (2011). Accelerated Profile HMM Searches. PLoS Comput Biol.

[53] Artimo P, Jonnalagedda M, Arnold K, Baratin D, Csardi G, de Castro E (2012). ExPASy: SIB bioinformatics resource portal. Nucleic Acids Res.

[54] Kapustin Y, Souvorov A, Tatusova T, Lipman D (2008). Splign: algorithms for computing spliced alignments with identification of paralogs. Biol Direct.

[55] Edgar RC (2004). MUSCLE: a multiple sequence alignment method with reduced time and space complexity. BMC bioinformatics.

[56] Stamatakis A, Hoover P, Rougemont J (2008). A rapid bootstrap algorithm for the RAxML Web servers. Syst Biol.

[57] Petersen TN, Brunak S, von Heijne G, Nielsen H (2011). SignalP 4.0: discriminating signal peptides from transmembrane regions. Nat Methods.

[58] Nei M, Gojobori T (1986). Simple methods for estimating the numbers of synonymous and nonsynonymous nucleotide substitutions. Mol Biol Evol.

[59] Tamura K, Peterson D, Peterson N, Stecher G, Nei M, Kumar S (2011). MEGA5: molecular evolutionary genetics analysis using maximum likelihood, evolutionary distance, and maximum parsimony methods. Mol Biol Evol.

[60] Friz CT (1967). The Biochemical composition of the free-living Amoebae Chaos Chaos, Amoeba dubia and Amoeba proteus. Comp Biochem Physiol.

[61] Parfrey LW, Lahr DJ, Katz LA (2008). The dynamic nature of eukaryotic genomes. Mol Biol Evol.

