# Supplementary material for: Protein import complexes in the mitochondrial outer membrane of Amoebozoa representatives
Source: BMC Genomics. 2016 Feb 6;17:99. doi: 10.1186/s12864-016-2402-2 (PMC4744386; doi:10.1186/s12864-016-2402-2)
Supplement: Additional file 1: Table S1. — Contains a table with the information on the reference sequences used as queries in tBLASTn searches against the transcriptome of A. castelanii, alignments of the identified subunits of theTOM, TOB/SAM and ERMES complexes in and their counterparts deposited in the GenBank and displaying differences in amino acid sequences, phylogenetic tress of the identified subunits, and graphical representation of the intron – exon gene structure for the identified subunits. (PDF 2670 kb) [file 12864_2016_2402_MOESM1_ESM.pdf]

Table S1

References for subunits of the TOM, TOB/SAM and ERMES complexes used in BLAST search.

Protein	Accession number and organism
Tom5	<u>Fungi</u>: EGX93488 *C. militaris*, EAA35832 *N. crassa*, EMS22290 *R. toruloides*, EDN61253 *S. cerevisiae*, G2TRP7 *S. pombe*<br><u>Viridiplantae</u>: NP_196421 *A. thaliana*, EDP08420 *C. reinhardtii*, EOY22084 *T. cacao*, P08196 *S. lycopersicum*<br><u>Metazoa</u>*:* A8YXZ8 *B. taurus*, EGV92195 *C. griseus*, NP_001127956 *H. sapiens*, ADO28137 *I. furcatus*, ADO28786 *I. punctatus*, AFI37119 *M. mulatta*, B1AXP6 *M. musculus*, EPQ18733 *M. brandtii*, Q5R676 *P. abelii*, ELW69860 *T. chinensis*
Tom6	<u>Fungi</u>*:* EJP66545 *B. bassiana*, CCG25521 *C. orthopsilosis*, CBF82512 *E. nidulans*, EKD21409 *M. brunnea*, EFY91581 *M. acridum*, EDP48137 *N. fumigate*, AAK18811 *N. crassa*, EKV11557 *P. digitatum*, EEA18753 *P. marneffei*, CAA99236 *S. cerevisiae*<br><u>Metazoa:</u> AAW82111 *B. taurus*, BAG30996 *H. sapiens*, AFE76132 *M. mulatta*, NP_079641 *M. musculus*, ELK00312 *P. alecto*, ELW66052 *T. chinensis*<br><u>Viridiplantae:</u> NP_564545 *A. thaliana*, EEE85918 P. *trichocarpa*
Tom7	<u>Amoebozoa</u>: Q54RZ5, *D. discoideum*, EFA78398 *P. pallidum*<br><u>Fungi</u>: A1CM16 *A. clavatus*, Q3MPF4 *C. albicans*, E3QAN6 *C. graminicola*, C8V0I0 *E. nidulans*, E5ABA6 *L. muculans*, Q9C1J0 *N. crassa*, A1DM09 *N. fischeri*, B6QV75 *P. marneffei*, A6ZS10 *S. cerevisiae*, B6K4E6 *S. japonicus*, CBQ67540 *S. reilianum*, F2PV03 *T. equinum*<br><u>Metazoa</u>: E2GEZ0 *B. taurus*, A8NG07 *B. malayi*, P34660 *C. elegans*, C1BNP2 *C. rogercresseyi*, E2AYL4 *C. floridanus*, B0X856 *C. quinquefasciatus*, Q7K036 *D. melanogaster*, C1BW89 *E. Lucius*, E2C311 *H. saltator*, Q75MR5 *H. sapiens*, C1BFD2 *O. mykiss*, C1BJ96 *O. mordax*, E0VJA3 *P. humanus subsp. Corporis*, D3ZMR1 *R. norvegicus*, B9EM79 *S. salar*, B5G498 *T. guttata*, E2J7F8 *T. matogrossensis*, A9JSP9 *X. tropicalis*<br><u>Opisthokonta</u>: E9C1N2 *C. owczarzaki*<br><u>Viridiplantae</u>: Q3ECI7 *A. thaliana*, A8IG88 *C. reinhardtii*, B9NKR7 *P. trichocarpa*, B9SC98 *R. communis*, O82067 *S. tuberosum*, Q6DL92 *T. aestivum*, B6SJR3 *Z. mays*
Tom20	<u>Fungi</u>: EER40887 *A. capsulatus*, CCG22491 *C. orthopsilosis*, EFW13694 *C. posadasii*, ACF06613 *E. guineensis*, CCA39581 *K. pastoris*, GAC72562 *P. antarctica*, EEH18555 *P. brasiliensis*, CCO31903 *R. solani*, EMS22925 *R. toruloides*, CAA97084 *S. cerevisiae*, EGD95676 *T. tonsurans*, CCH45357 *W. ciferrii*<br><u>Metazoa</u>: XP_001652747 *A. aegypti*, ADY43237 *A. suum*, NP_001092653 *B. taurus*, A8Y3V5 *C. briggsae*, Q19766 *C. elegans*, EMC86060 *C. livia*, EMP27268 *C. mydas*, EDS31058 *C. quinquefasciatus*, GAA35186 *C. sinensis*, EHJ74209 *D. plexippus*, NP_001002698 *D. rerio*, ACO14271 *E. lucius*, ADD20139 *G. morsitans*, ADD38509 *L. salmonis*, NP_077176 *M. musculus*, AAL05599 *M. unguiculatus*, NP_001135431 *N. vitripennis*, AFJ91791 *O. edulis*, ELK05097 *P. alecto*, NP_690918 *R. norvegicus*, ACM09147 *S. salar*, , ELW68132 *T. chinensis*, AAH76705 *X. (Silurana) tropicalis*, NP_001080323 *X. laevis*<br><u>Stramenopiles:</u> CBJ27905 *E. siliculosus*<br><u>Viridiplantae</u>: EMT00308 *A. tauschii*, AAK64184 *A. thaliana*, ABF74690 *H. brasiliensis*, AES72042 *M. truncatula*, A2WYG9 *O. sativa*, XP_003082381 *O. tauri*, CAA63223 *S. tuberosum*, EMS57714 *T. urartu*, AFW84136 *Z. mays*, ACG30214 *Z. mays*
Tom22	<u>Fungi</u>: EER37412 *H. capsulatus*, XP_002620115 *B. dermatitidis*, XP_003017458 *A. benhamiae*, XP_001269087 *A. clavatus*, KEY79581 *A. fumigatus*, CAE47910 *A. fumigatus*, XP_710764 *C. albicans*, XP_001239444 *C. immitis*, XP_003196226 *C. gattii*, XP_384291 *F. graminearum*, EFX03100 *G. clavigera*, XP_002491829 *K. pastoris*, XP_956695 *N. crassa*, XP_001264575 *N. fischeri*, EEH38491 *P. lutzii*, P49334 *S. cerevisiae*, O13813 *S. pombe*, EGD96435 *T. tonsurans*, EGY19429 *V. dahliae*<br>**Choanoflagellida**: EGD79841 *S. rosetta*<br><u>Metazoa</u>: XP_003221021 *A. carolinensis*, DAA34785 *A. variegatum*, AAI49340 *B. taurus*, CCD65583 *C. elegans*, XP_852619 *C. l. familiaris*, EFN74091 *C. floridanus*, EGW01687 *C. griseus*, GAA56922 *C. sinensis*, AAF241222 *D. melanogaster*, AAI53451 *D. rerio*, ADD18896 *G. m. morsitans*, EHB02205 *H. glaber*, EFN83430 *H. saltator*, BAB16408 *H. sapiens*, XP_003419809 *L. africana*, XP_010709228 *M. gallopavo*, NP_064628 *M. mulatta*, NP_766197 *M. musculus*, AES08601 *M. furo*, XP_003264803 *N. leucogenys*, ACO09752 *O. mordax*, XP_002831181 *P. abelii*, EEB15197 *P. humanus*, XP_001163726 *P. troglodytes*, NP_997679 *R. norvegicus*, XP_003771053 *S. harrisii*, ACM08673 *S. salar*, XP_003126064 *S. scrofa*, NP_001039252 *X. tropicali*<br><u>Virdiplantae:</u> XP_002892196 *A. lyrata*, XP_003078090 *O. tauri*, NP_001241795 *Z. mays*, XP_002301850 *P. trichocarpa*, XP_002272982 *V. vinifera*
Tom40	<u>Alveolata:</u> CAG24986 *P. falciparum*<br><u>Amoebozoa</u>: ADZ24223 *A. castellanii*, XP_642798 *D. discoideum*, XP_004352318 *D. fasciculatum*, EFA80126 *P. pallidum*<br><u>Cryptophyta:</u> EKX33332 *G. theta CCMP2712*<br><u>Fungi</u>: Q5AH14 *C. albicans*, AET97826 *N. bombycis*, P24391 *N. crassa*, W6QMH2 *P. roqueforti*, P23644 *S.cerevisiae*, O13656 *S. pombe*<br><u>Haptophyceae</u>: EOD14807 *E. huxleyi CCMP1516*<br><u>Metazoa</u>: Q18090 *C. elegans*, Q9U4L6 *D. melanogaster*, A1Z6L1 *D. melanogaster*, O96008 *H. sapiens*, BAL42839 *M. crassicauda*, B5X251 *S. salar*, B5X9H3 *S. salar*, Q6P825 *X. tropicalis*<br><u>Rhodophyta:</u> BAM79444 *C. merolae strain 10D*<br><u>Stramenopiles:</u> CBJ27101 *E. siliculosus*, EWM29712 *N. Gaditana*<br><u>Trimastix:</u> ABW76113 *T. pyriformis*<br><u>Virdiplantae:</u> KFM28529 *A. protothecoides*, Q9LHE5 *A. thaliana*, Q9SX55 *A. thaliana*, CCO66139 *B. prasinos*, XP_008453929 *C. melo*, EDP06354 *C. reinhardtii*, EFN52297 *C. variabilis*, NP_001242605 *G. max*, KDD73653 *Helicosporidium sp. ATCC 50920*, XP_008359999 *M. domestica*, ACO68486 *M. sp. RCC299*, XP_006644056 *O. brachyantha*, ABO95318 *O. lucimarinus CCE9901*, Q10M45 *O. sativa subsp. japonica*, XP_008779040 *P. dactylifera*, XP_008790463 *P. dactylifera*, XP_008792477 *P. dactylifera*, XP_008221088 *P. mume*, JAC84130 *Tetraselmis sp. GSL018*, EFJ44900 *V. carteri f. nagariensis*, XP_008655478 *Z. mays*, XP_008658595 *Z. mays*
Tom70	<u>Fungi</u>: CCG25000 *C. orthopsilosis*, EIF49158 *D. bruxellensis*, AAO32536 *N. castellii*, AAD21979 *N. crassa*, EAL87263 *N. fumigate*, CAA75047 *P. anserina*, ABN58618 *S. cerevisiae*, O14217 *S. pombe*<br><u>Metazoa</u>: EGI57310 *A. echinatior*, NP_001068796 *B. taurus*, EKC37920 *C. gigas*, AAF53148 *D. melanogaster*, O94826 *H. sapiens*, AAI39421 *M. musculus*, BAD11366 *R. norvegicus*<br><u>Stramenopiles</u>: CBK23055 *B. hominis*, ADL28121 *B. sp. NandII*, CBJ31076 *E. siliculosus*, AA032537 *N. castellii*
Metaxin1	<u>Metazoa</u>: EGI63220 *A. echinatior*, NP_001156282 *A. pisum*, EDP36824 *B. malayi*, ELR54251 *B. mutus*, A8XWD1 *C. briggsae*, O45503 *C. elegans*, EFN67703 *C. floridanus*, EKC20634 *C. gigas*, Q9VHB6 *D. melanogaster*, NP_001007281 *D. rerio*, EHB07315 *H. glaber*, Q13505 *H. sapiens*, EAW53108 *H. sapiens*, NP_942584 *H. sapiens*, Q4R3I0 *M. fascicularis*, AFE70029 *M. mulatta*, NP_001155296 *M. musculus*, NP_038632 *M. musculus*, P47802 *M. musculus*, EDLA15228 *M. musculus*, AES02372 *M. putorius furo*, XP_003701385 *M. rotundata*, ELK02789 *P. alecto*, EEB16319 *P. humanus*, XP_003308483 *P. troglodytes*, NP_001094137 *R. norvegicus*, EDM00659 *R. norvegicus*, ACI33431 *S. salar*, BAF91494 *S. scrofa*, NP_001084470 *X. laevis*, AAI35683 *X. tropicalis*

**Metaxin2**	**Metazoa**: NP_001094710 *B. taurus*, ABC33802 *C. elegans*, ACO10187 *C. rogercresseyi*, ACU32593 *C. milii*, EFN60236 *C. floridanus*, EKC43002 *C. gigas*, EGW02671 *C. griseus*, AFJ50577 *C. adamanteus*, AAP96761 *D. rerio*, EFN76553 *H. saltator*, EAX11078 *H. sapiens*, EAX11076 *H. sapiens*, EAX11077 *H. sapiens*, ADO28229 *I. furcatus*, ADO29224 *I. punctatus*, EFO23963 *L. loa*, AFP99270 *L. intermedia*, AFE64149 *M. mulatta*, AAY21064 *M. musculus*, AES02373 *M. putorius furo*, ELK32717 *M. davidii*, ELK04731 *P. alecto*, EDL79192 *R. norvegicus*, ACI69201 *S. salar*, NP_001134863 *S. salar*, NP_001038006 *S. scrofa*, ACH43784 *T. guttata*, NP_001098699 *T. rubripes*, ELW64130 *T. chinensis*, NP_001084472 *X. laevis*, AAT57871 *X. tropicalis*
**Tob38/ Sam35**	**Fungi**: EAL90650 *A. fumigatus*, CBF89045 *A. nidulans*, CCD44290 *B. cinerea*, CCG24718 *C. orthopsilosis*, EFY90049 *M. acridum*, EAW22545 *N. fischeri*, P14693 *S. cerevisiae*, EFE39159*T. verrucosum*<br>**Stramenopiles:** CBJ32432 *E. siliculosus*
**Mas37/ Sam37**	**Fungi**: EAW11375 *A. clavatus*, GAD92478 *B. spectabili*, EAL03688 *C. albicans*, XP_002418173 *C. dubliniensis*, EFW15805 *C. posadasii*, EXV02056 *M. robertsii*, EAW18120 *N. fischeri*, EKV09579 *P. digitatum*, P50110 *S. cerevisiae*, EED15167 *T. stipitatus*<br>**Metazoa**: ADD20070 *G. morsitans*
**Tob55/ Sam50**	**Amoebozoa**: model from: Wojtkowska et al. 2010 from *A. castellanii*<br>**Fungi:** EED44828 *A. flavus NRRL3357*, EAL84610 *A. fumigatus Af293*, CBF79308 *A. nidulans FGSC A4*, CCD45409 *B. cinerea T4*, CCG25583 *C. orthopsilosis*, CBY00086 *L. maculans, JN3*AAS76651 *N. crassa*, EKV15900 *P. digitatum PHI26*, EDN62785 *S. cerevisiae YJM789*, EPY52358 *S. cryophilus OY26*, EEB06890 *S. japonicus yFS275*, EPX73056 *S. octosporus yFS286*, CAA97352 *S. pombe*, ABN68113 *S. stipitis CBS 6054*, EEA24609 *T. marneffei ATCC 18224*, EED18370 *T. stipitatus ATCC 10500*<br>**Metazoa:** JAB65435 *A. glabripennis*, JAC44697 *B. dorsalis*, JAB85363 *C. capitata*, AAC72375 *D. melanogaster*, Q9V784 *D. melanogaster*, Q9Y512 *H. sapiens*, JAA88618 *P. aegeria*, EEB19878 *P. humanus corporis*, CCD81062 *S. mansoni*, ACI66168 *S. salar*, ACI33501 *S. salar*, NP_001083329 *X. laevis*<br>**Stramenopiles:** CBJ33514 *E. siliculosus*<br>**Viridiplantae:** EMT14957 *A. tauschii*, EEF48875 *R. communis*, EEF41943 *R. communis*, EEF34085 *R. communis*, EMS50338 *T. urartu*
**Mdm10**	**Fungi**: EJP66870 *B. bassiana*, C4YIG3 *C. albicans*, EAU84591 *C. cinerea*, ADV25733 *C. gattii*, ELA31908 *C. gloeosporioides*, AAW47220 *C. neoformans*, CCG21274 *C. orthopsilosis*, CCE32471 *C. purpurea*, CCA36709 *K. pastoris*, CAA75046 *P. anserina*, AAC04947 *S. cerevisiae*, DAA06978 *S. cerevisiae*, CAE00796 *S. macrospora*, ELU45447 *T. cucumeris*, EEY14339 *V.alfalfae*, EGY13374 *V. dahliae*
**Mdm12**	**Fungi**: C0NF00 *A. capsulatus*, A1CNY1 *A. clavatus*, C5GK63 *A. dermatitidis*, B8MZJ8 *A. flavus*, Q75CC2 *A. gossypii*, Q5BF59 *A. nidulans*, A2QAU8, *A. niger*, Q2UQG9 *A. oryzae*, C5FUT6 *A. otae*, Q0CZL5 *A. terreus*, A6RLP9 *B. fuckeliana*, Q59S52 *C. albicans*, A8NEF5 *C. cinerea okayama*, B9WK07 *C. dubliniensis*, Q6FVF2 *C. glabrata*, Q2HDE7 *C. globosum*, Q1E2F1 *C. immitis*, C4YBT3 *C. lusitaniae*, B5RUL6 *D. hansenii*, Q6CUC3 *K. lactis*, C4R415 *K. pastoris*, B0DQ09 *L. bicolor*, A5E771 *L. elongisporus*, C5DEN8 *L. thermotolerans*, A8PSC0 *M. globosa*, A5DAR4 *M. guilliermondii*, A4RM00 *M. oryzae*, Q7SEZ6 *N. crassa*, A1D1T8 *N. fischeri*, Q4WRX2 *N. fumigate*, B2AA87 *P. anserine*, B6HAR6 *P. chrysogenum*, C1H3V1 *P. lutzii*, B6QAV0 *P. marneffei*, Q0U1X7 *P. nodorum*, B8P9E4 *P. placenta*, B2W543 *P. tritici-repentis*, B3LJ47 *S. cerevisiae*, B6K4Z5 *S. japonicas*, Q92377 *S. pombe*, A7ELE2 *S. sclerotiorum*, A3LWH1 *S. stipites*, XP_003232252 *T. rubrum*, B8M2V6 *T. stipitatus*, Q4PEB4 *U. maydis*, C4JY59 *U. reesii*, A7TFP8 *V. polyspora*, C5DTC4 *Z. rouxii*
**Mdm34/ Mmm2**	**Fungi**: C0NY51 *A. capsulatus*, C5JVU4 *A. dermatitidis*, C5FRB0 *A. otae*, A1CHU1 *A. clavatus*, A2QJH4 *A. niger*, Q2UJF5 *A. oryzae*, Q0CZY7 *A. terreus*, A6S9E2 *B. fuckeliana*, C4YE34 *C. albicans*, B9W824 *C. dubliniensis*Q6FQE0 *C. glabrata* CCG21585 *C. orthopsilosis*, Q2H9Y1 *C. globosum*, C4Y6F4 *C. lusitaniae*, Q1E4T3 *C. immitis*, A8NYS9 *C. cinerea*, P0CO71 *C. neoformans*, Q5BBM5 *E. nidulans*, C4QYM9 *K. pastoris*, B0CXH5 *L. bicolor*, A5DUL5 *L. elongisporus*, A4QT54 *M. oryzae*, A8QD14 *M. globosa*, A5DLK4 *M. guilliermondii*, A1CWW9 *N. fischeri*, B0Y6I4 *N. fumigate*, Q7RZK9 *N. crassa*, C0S713 *P. brasiliensis*, B6QIB3 *P. marneffei*, B2VS19 *P. tritici-repentis*, EJS43835 *S. arboricola*, B3LHR1 *S. cerevisiae*, A7EWF5 *S. sclerotiorum*, B8MKT9 *T. stipitatus*, C4JQ45 *U. reesii*, Q4PFA7 *U. maydis*, Q6C7W0 *Y. lipolytica*
**Mmm1**	**Fungi**: EEH03033 *A. capsulatus*, EFR03467 *A. gypseum*, GAA86306 *A. kawachii*, EJP62622 *B. bassiana*, CCG23843 *C. orthopsilosis*, CCA40584 *K. pastoris*, EDK46090 *L. elongisporus*, AAF43713 *N. crassa*, EDP47543 *N. fumigate*, EEH50174 *P. brasiliensis*, EDU40624 *P. tritici-repentis*, CAA97449 *S. cerevisiae*, EEB09187 *S. japonicas*, BAA21458 *S. pombe*
**Gem1**	**Amoebozoa:** XP_647338 D. *discoideum*, EGG25101 *D. fasciculatum*, F1A505 *D. purpureum*, EFA85557 *P. pallidum*<br>**Fungi:** Q758X6 *A. gossypii*, Q2UM43 *A. oryzae*, Q5ABR2 *C. albicans*, Q6FIR8 *C. glabrata*, P0CO78 *C. neoformans*, Q5B5L3 *E. nidulans*, Q4I2W2 *G. zeae*, XP_006966705 *T. reesei* QM6a, Q6CY37 *K. lactis*, Q7RZA2 *N. crassa*, Q4WN24 *N. fumigata*, W6QF87 *P. roqueforti*, P39722 *S. cerevisiae*, O59781 *S. pombe*, Q4PB75 *U. maydis*, Q6C2J1 *Y. lipolytica*<br>**Metazoa:** Q94180 *C. elegans*, Q8IMX7 *D. melanogaster*, Q298L5 *D. pseudoobscura pseudoobscura*, Q6NVC5 *D. rerio*, Q5ZM73 *G. gallus*, Q5ZM83 *G. gallus*, Q8IXI2 *H. sapiens*, Q8IXI1 *H. sapiens*, Q8BG51 *M. musculus*, Q7TSA0 *R. norvegicus*, XP_791124 *S. purpuratus*, Q864R5 *S. scrofa*, Q6DIS1 *X. tropicalis*<br>**Opisthokonta**: EFW47272 *C. owczarzaki*<br>**Viridiplantae:** Q8RXF8 *A. thaliana*, EFJ25633 *S. moellendorffii*

Figure S1

Alignments of the identified subunits of the TOM, TOB/SAM and ERMES complexes in and their counterparts deposited in the GenBank and displaying differences in amino acid sequences.

Red background of letters denotes indels whereas conservative and radical substitutions are marked by blue and green background, respectively.

A. castellanii Tom70

```
Tom70_XP_004339622.1      MEAKVKEANALFLQGKHSPAVALYTAAIEA-----------GAAYLRLGLYRKCLADCEA
Tom70_Ac_transcriptome    MEAKVKEANALFLQGKHSPAVALYTAAIEAGSPTATLLCNRGAAYLRLGLYRKCLADCEA
                          ******************************           *******************

Tom70_XP_004339622.1      ALRLQPADPRPYLLKGKALVGMNKSADAEAAWRAGLDKADGAADVELLLQLQGQLNPPVA
Tom70_Ac_transcriptome    ALRLQAADPRPYLLKGKALVGMNKSADAEAAWRAGLDKADGAADVELLLQLQGQLNPPVA
                          *****.******************************************************

Tom70_XP_004339622.1      APAIATAPAASSEPKPAVITNGEAKATITTPSPQASAVPRAAEPAGKTKSPAPAQQPKKE
Tom70_Ac_transcriptome    APAIATAPAASSEPKPAVITNGE--ATITTPSPQASAVPRAAEPAGKTKSPAPAQQPKKE
                          ***********************  ***********************************

Tom70_XP_004339622.1      AKSPPTAAAVAAANPNDLAEASAMVAARGLVQHGSGNTTLDEKIAMGYLHVNTGNFPQAI
Tom70_Ac_transcriptome    AKSPPTAAAVAAANPNDLAEASAMVAARGLVQHGSGNTTLDEKIAMGYLHVNTGNFPQAI
                          ************************************************************

Tom70_XP_004339622.1      KLFNVLVNLYPKLVAAYLGRGTAYALSGHLSTAVEEFSAAIKIDDTCMEAWKRRGQSRAA
Tom70_Ac_transcriptome    KLFNVLVNLYPKLVAAYLGRGTAYALSGHLSTAVEEFSAAIKIDDTCMEAWKRRGQSRAA
                          ************************************************************

Tom70_XP_004339622.1      MGQDAEAVLDLTRAAELAPKDADIYHQRGLIYFKLRNYGRAAEDFRRATAADAMSKLSWN
Tom70_Ac_transcriptome    MGQDTEAVLDLTRAAELAPKDADIYHQRGLIYFKLRNYGRAAEDFRRATAADAMSKLSWN
                          ****:*******************************************************

Tom70_XP_004339622.1      HLGLCLNALGRPMEAIQAHKRALELDPAFREALANIGQAYKDYGNSLKAEKYFAKGLKVD
Tom70_Ac_transcriptome    HLGLCLNALGRPMEAIHAHKRALELDPAFREALANIGQAYKDYGNSLKAEKYFAKGLKVD
                          ****************:*******************************************

Tom70_XP_004339622.1      PNYMHAFHLRGLARFGAGDHRGALSDFTAALRVDDKHKDSRLMRGIVLHGLGRFREAVAD
Tom70_Ac_transcriptome    PNYMHAFHLRGLARFGAGDHRGALSDFTAALRVDDKHKDSRLMRGIVLHGLGRFREAVAD
                          ************************************************************

Tom70_XP_004339622.1      YDVLVREKPDHVAWYNRQIALWTHHHLDTPVAHFNIDRVLNAYFKEAWCKRLDPATLTSY
Tom70_Ac_transcriptome    YDVLVREKPDHVAWYNRQIALWTHHHLDTPVAHFNIDRVLNAYFKEAWCKRLDPATLTSY
                          ************************************************************

Tom70_XP_004339622.1      TSQPPINNAIADVALNDELRSEHAKLLIRAAVDIGKKIQLNCPGYLANQRQQRACGFAII
Tom70_Ac_transcriptome    TSQPPINNAIADVALNDELRSEHAKLLIRAAVDIGKKIQLNCPGYLANQRQQRACGFAII
                          ************************************************************

Tom70_XP_004339622.1      ELAQTLRRVWAGEETQLSGKASSLTDQPHTFAWRDLYDIPIRWRQFSEPNDPVWWVDLLS
Tom70_Ac_transcriptome    ELAQTLRRVWAGEETQLSGKASSLTDQPHTFAWRDLYDIPIRWRQFSEPNDPVWWVDLLS
                          ************************************************************

Tom70_XP_004339622.1      KTLHEAVQHDFWVVTPCHSLARPNRIMEGTRLTIQRSPPEGYEFSIRTPGTPNRWQEYNA
Tom70_Ac_transcriptome    KTLHEAVQHDFWVVTPCHSLARPNRIMEGTRLTIQRSPPEGYEFSIRTPGTPNRWQEYNA
                          ************************************************************

Tom70_XP_004339622.1      EMAHNYRLLQEEASKPHRDLSKLTELVLHMAFYWYNFMPLSRGTAAVGLVTVHAMFLALG
Tom70_Ac_transcriptome    EMAHNYRLLQEEASKPDRDQSKLTELVLHMAFYWYNFMPLSRGTAAVGLVTVHAMFLALG
                          **************** ** ****************************************

Tom70_XP_004339622.1      FEMESGLPQGLQPDWEGILTARPSDFVKCLRSAWIDAACRPTTTLDALPLVAQVCPTLRH
Tom70_Ac_transcriptome    FEMESGLPQGLQPDWEGILTARPSDFVRCLRSAWIDAACRPTTTLDALPLVAQVCPTLRH
                          ***************************.********************************

Tom70_XP_004339622.1      MVLALNAAS
Tom70_Ac_transcriptome    MVLALNAAS
                          *********
```

Figure S1A

A. castellanii Tom22 A

```
Tom22_XP_004353494.1      --------------------------MPDPHRYMPHSFLKVDTTRLDQWRPALDDAATDA
Tom22_Ac_transcriptome    MARPSHHHEFLLLLAVAFLSVGLARQMPDPHRYMPHSFLKVDTTRLDQWRPALDDAATDA
                                                    **********************************

Tom22_XP_004353494.1      VRVINSHRPRDQHLALDQVQTDGVQVGRALAFSGTIELADHHHPPHPSAYTFTQHSDFIH
Tom22_Ac_transcriptome    VRVINSHRPRDQHLALDQVQTDGVQVGRALAFSGTIELADHHHPPHPSAYTFTQHSDFIH
                          ************************************************************

Tom22_XP_004353494.1      LQPVQPLAPLWALPSPSASLKTEPVDQLPQPDEDPVFPEVELEGPLVLHLDQQLYDPVLR
Tom22_Ac_transcriptome    LQPVQPLAPLWALPSPSASLKTEPVDQLPQPDEDPVFPEVELEGPLVLHLDQQLYDPVLR
                          ************************************************************

Tom22_XP_004353494.1      LPHSVEVGAVRVIRVGPGVRLRVGGVSQMKLRSSLDVQRNIKLLALMDENNATTANLHQQ
Tom22_Ac_transcriptome    LPHSVEVGAVRVIRVGPGVRLRVGGVSQMKLRSSLDVQRNIKLLALMDENNATTANLHQQ
                          ************************************************************

Tom22_XP_004353494.1      GEMDVPELELKLVDQGRAFRMWSSSAERVKAKRRSAHVLELLPTAASPSADVSSQVTTTS
Tom22_Ac_transcriptome    GEMDVPELELKLVDQGRAFRMWSSSAERVKAKRRSAHVLELLPTAASPSADVSSQVTTTS
                          ************************************************************

Tom22_XP_004353494.1      HGRTDLAPRSASDASLWVLSQANLLRGVIGAFAQAAEEQPVVLRRVVEAKAEAVSVLVIP
Tom22_Ac_transcriptome    HGRTDLAPRSASDASLWVLSQANLLRGVIGAFAQAAEEQPVVLRRVVEAKAEAVSVLVIP
                          ************************************************************

Tom22_XP_004353494.1      MTLREQPHNNHGGKTSQWQVVVTRGADGRHTVVSHQQLVQQESHVTVAPWVWANTTTPAV
Tom22_Ac_transcriptome    MTLREQPHNNHGGKTSQWQVVVTRGADGRHTVVSHQQLVQQESHVTVAPWVWANTTTPAV
                          ************************************************************

Tom22_XP_004353494.1      MRTVQLLEKNAHRPAQASS
Tom22_Ac_transcriptome    MRTVQLLEKNAHRPAQASS
                          *******************
```

Figure S1B

A. castellanii Tom22 B

```
Tom22_XP_004358239.1        --------------------------MPDPHRYMPHSFLKVDTTRLDQWRSALDDAATDA
Tom22_Ac_transcriptome      MARTSHHHEFLLLLIVAFLYVGLARQMPDPHRYMPHSFLKVDTTRLDQWRSALDDAATDA
                                                      **********************************

Tom22_XP_004358239.1        VRVLNSHRPRDQHLALDQVQTDGVQVGRALAFSGTIELADHHHPPHPSAYTFTQHSDFIH
Tom22_Ac_transcriptome      VRVLNSHRPRDQHLALDQVQTDGVQVGRALAFSGTIELADHHHPPHPSAYTFTQHSDFIH
                            ************************************************************

Tom22_XP_004358239.1        LQPVQPLSPLWALPSPSASLKTEPVYQLPQPDEDPVFP
Tom22_Ac_transcriptome      LQPVQPLSPLWALPSPSASLKTEPVYQLPQPDEDPVFP
                            **************************************
```

Figure S1C

A. castellanii Metaxin

```
Metaxin_XP_004337900.1       MASSSSSAAPAVLKLHQYGARWELPSFDPFCLSAQAYMRLAGVTFEE-------------
Metaxin_Ac_transcriptome     MASSSSSAAPAVLKLHQYGARWELPSFDPFCLSAQAYMRLAGVTFEEVPSNNPDVSPTSN
                             ***********************************************             

Metaxin_XP_004337900.1       -----LGDDYVAGTNAIFTYVGNKTGKSLDSALNAEQKATAAAFIHLIETKLHPTLLYNW
Metaxin_Ac_transcriptome     LPLVQLGDDYVAGTNAIFTYVGNKTGKSLDSALNAEQKATAAAFIHLIETKLHPTLLYNW
                                  *******************************************************

Metaxin_XP_004337900.1       WAEKQNMGQTLVLPHFNSMVFPLGYVLPRLKQRNVQSYLYTLNLTQDEKVYNDAEECYAA
Metaxin_Ac_transcriptome     WAEKQNMGQTLVLPHFNSMVFPLGYVLPRLKQRNVQSYLYTLNLTQDEKVYNDAEECYAA
                             ************************************************************

Metaxin_XP_004337900.1       LADFLGDKHFFFGD---SLDAVAFGHLAIHLVAPQSHKLRSRLLQHKNLEAFCKRVMTLY
Metaxin_Ac_transcriptome     LADFLGDKHFFFGDSPSSLDAVAFGHLAIHLVAPQSHKLRSRLLQHKNLEAFCKRVMTLY
                             **************   *******************************************

Metaxin_XP_004337900.1       FGQDFPAIPAPPPATEDKDQKQEMSQHKKTGMYLLTGAGLLILLHYLTKQHQAAHQ
Metaxin_Ac_transcriptome     LGQDFPAIPAPPPATEDKDQKQEMSQHKKTGMYLLTGAGLLILLHYLTKQHQAAHQ
                             :*******************************************************
```

Figure S1D

A. castellanii Tob55/Sam50

```
Tob55/Sam50_XP_004341043.1        MSYTDEDEPIDLGVRGEDAWRVRRVWVKGNRRTRPDVVAACVKPVLKARTFDEVLARVAE
Tob55/Sam50_Ac_transcriptome      MSYTDEDEPIDLGVRGEDAWRVRRVWVKGNRRTRPDVVAACVKPVLKARTFDEVLARVAE
                                  ************************************************************

Tob55/Sam50_XP_004341043.1        AAGELKGLGIFKSVNLVLDDLPDDVAAADPSACDLRVELVEHKLTRIEVKTSTTVGENEP
Tob55/Sam50_Ac_transcriptome      AAGELKGLGIFKSVNLVLDDLPDDVAAADPSACDLRVELVEHKLTRIEVKTSTTVGENEP
                                  ************************************************************

Tob55/Sam50_XP_004341043.1        DVQATVGLCNAFGRAETVSVSAQVGASNWREHWSSAFSLAFKRP----------------
Tob55/Sam50_Ac_transcriptome      DVQATVGLCNAFGRAETVSVSAQVGASNWREHWSSAFSLAFKRPVLGQGPGPRHIEADAT
                                  ********************************************

Tob55/Sam50_XP_004341043.1        ----------------------------VSYEASARHVIPESSAPLALRSHAGHSLKSAV
Tob55/Sam50_Ac_transcriptome      RASHRLPWCALTQTTHGLALRYSLPSHQVSYEASARHVIPESSAPLALRSHAGHSLKSAV
                                                              ********************************

Tob55/Sam50_XP_004341043.1        KYVYAHDTRDDPLTPTTGHAFTSSTELAGLGGDVRFVKQEMAAQLNLPLGKTRCSFNVLA
Tob55/Sam50_Ac_transcriptome      KYVYAHDTRDDPLTPTTGHAFTSSTELAGLGGDVRFVKQEMAAQLNLPLGKTRCSFNVLA
                                  ************************************************************

Tob55/Sam50_XP_004341043.1        KAGYLHALDSGSRI--------------------------RALGGGAYWAGSLHLSFPLP
Tob55/Sam50_Ac_transcriptome      KAGYLHALDSGSRIVDRFFLGGPGSIRGFQYNAVGPSHQQRALGGGAYWAGSLHLSFPLP
                                  **************                          ********************

Tob55/Sam50_XP_004341043.1        LRDVPDFVSGHLFANAGNLRQPTPGRSVAANVQDLFSADDVRAAVGAGLVLRTMFGRVEL
Tob55/Sam50_Ac_transcriptome      LRDVPDFVSGHLFANAGNLRQPTPGRSVAANVQDLFSADDVRAAVGAGLVLRTMFGRVEL
                                  ************************************************************

Tob55/Sam50_XP_004341043.1        NLSHPIRKAATDLVQPFQVGLSVRFL
Tob55/Sam50_Ac_transcriptome      NLSHPIRKAATDLVQPFQVGLSVRFL
                                  **************************
```

Figure S1E

A. castellanii Gem1

```
Gem1_XP_004356731.1         MKDQVRIVLIGDDGAGKTSLIATLLADSFQEV----------------------------
Gem1_Ac_transcriptome       MKDQVRIVLIGDDGAGKTSLIATLLADSFQEVVEQVVPELTIPPSVTNDRVSTHIIDTSS
                            ********************************                            

Gem1_XP_004356731.1         ---HKMDQQLRTADVVCLCYAADSENVAERITHWLRHIRHTRQECRAPQVPVILVGNKID
Gem1_Ac_transcriptome       RFQHKMDQQLRTADVVCLCYAADSENVAERITHWLRHIRHTRQECRAPQVPVILVGNKID
                               *********************************************************

Gem1_XP_004356731.1         LRGEDLTNPKLQEDMEPIMEEFKEVETCIECSAKSFLNVHEVFYFAQRAVLYPTAVLYDA
Gem1_Ac_transcriptome       LRGEDLTNPKLQEDMEPIMEEFKEVETCIECSAKSFLNVHEVFYFAQRAVLYPTAVLYDA
                            ************************************************************

Gem1_XP_004356731.1         GSRSLREECVAALKRIFKLCDKDRDGILSDDELNAFQARCFGASLDPAELQGVKDVVRGN
Gem1_Ac_transcriptome       GSRSLREECVAALKRIFKLCDKDRDGILSDDELNAFQARCFGASLDPAELQGVKDVVRGN
                            ************************************************************

Gem1_XP_004356731.1         VENGLTEKGLTLTGFLFLHHLFIQKGRLETTWTVLRQFGYDDNLRVDVASLCHSLPAPPP
Gem1_Ac_transcriptome       VENGLTEKGLTLTGFLFLHHLFIQKGRLETTWTVLRQFGYDDNLRVDVASLCHSLPAPPP
                            ************************************************************

Gem1_XP_004356731.1         SHVYELSQAGVAFFSSLHRAFDKGAGLALDDLDDLFSTAAPGLPALWTSGELDPATATQL
Gem1_Ac_transcriptome       SHVYELSQAGVAFFSSLHRAFDKGAGLALDDLDDLFSTAAPGLPALWTSGELDPATATQL
                            ************************************************************

Gem1_XP_004356731.1         NAAHISLRGWLALWSYTTVHDHKTTLEYLAWLGFESDPQLALALKPRREASALLCLVVGS
Gem1_Ac_transcriptome       NAAHISLRGWLALWSYTTVHDHKTTLEYLAWLGFESDPQLALALKPRREASALLCLVVGS
                            ************************************************************

Gem1_XP_004356731.1         PALTDAFLGDFLGKRDHEPSHATSTSITTTTRSPSSRLVALGSLPDISGTEKYVAMRKYG
Gem1_Ac_transcriptome       PALTDAFLGDFLGKRDHEPSHATSTSITTTTRSPSSRLVALGSLPDISGTEKYVAMRKYG
                            ************************************************************

Gem1_XP_004356731.1         LEETEALRTRLHEASLVIVLYDSSDSRSLSNTAHAGAELNSLCRNRDTPILHLALHHTAT
Gem1_Ac_transcriptome       LEETEALRTRLHEASLVIVLYDSSDSRSLSNTAHAGAELNSLCRNRDTPILHLALHHT--
                            **********************************************************  

Gem1_XP_004356731.1         LMLQQDGATVPDLSENGIHLEAFASYSTLLIKAHQLARREQEWQWLPTWPQAALLGAVAV
Gem1_Ac_transcriptome       -----DGATVPDLSENGIHLEAFASYSTLLIKAHQLARREQEWQWLPTWPQAALLGAVAV
                                 *******************************************************

Gem1_XP_004356731.1         LVGGILLYRHFKGSKQA
Gem1_Ac_transcriptome       LVGGILLYRHFKGSKQA
                            *****************
```

Figure S1F

Figure S2

Evolutionary analysis of the identified subunits based on Maximum Likelihood analysis. The RAxML rapid bootstrapping algorithm was used applying 1000 bootstrap replicated. All figures shows unrooted tree. Ac – *A. castellanii*, Ap – *A. proteus*, Dd – *D. discoideum*, Dp – *D. purpureum*, Df – *D. fasciculatum*, Pp – *P. pallidum*, Ed – *E. dispar*, En – *E. nuttalli.* Blue color indicates representatives of Discosea; red color indicates representatives of Tubulinea; green color indicates representatives of Mycetozoa; pink color indicates representatives of Archamoeba. Yellow line indicates Conosa group; light blue color indicates Lobosa.

Tom20

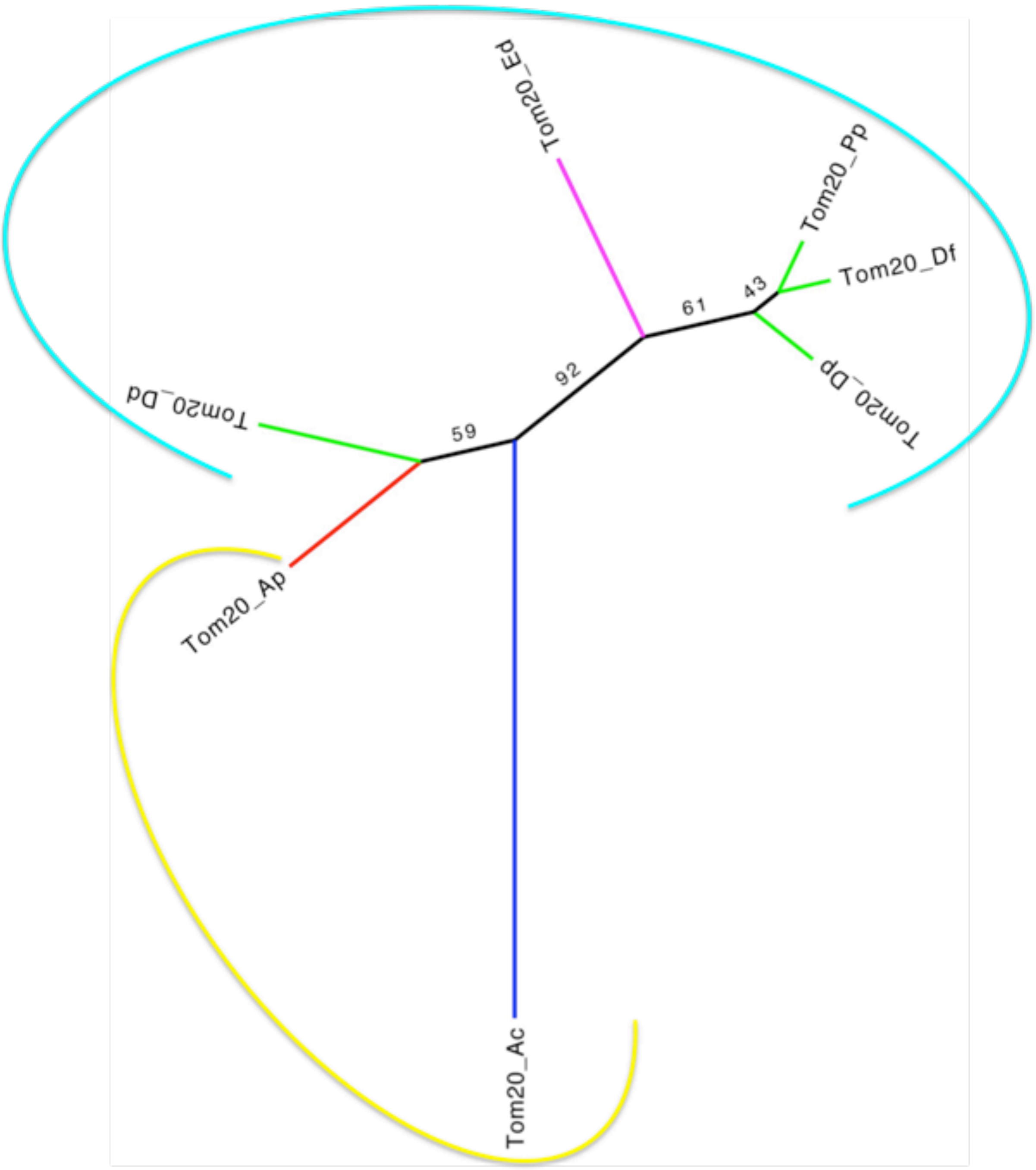

Figure S2A

Tom40

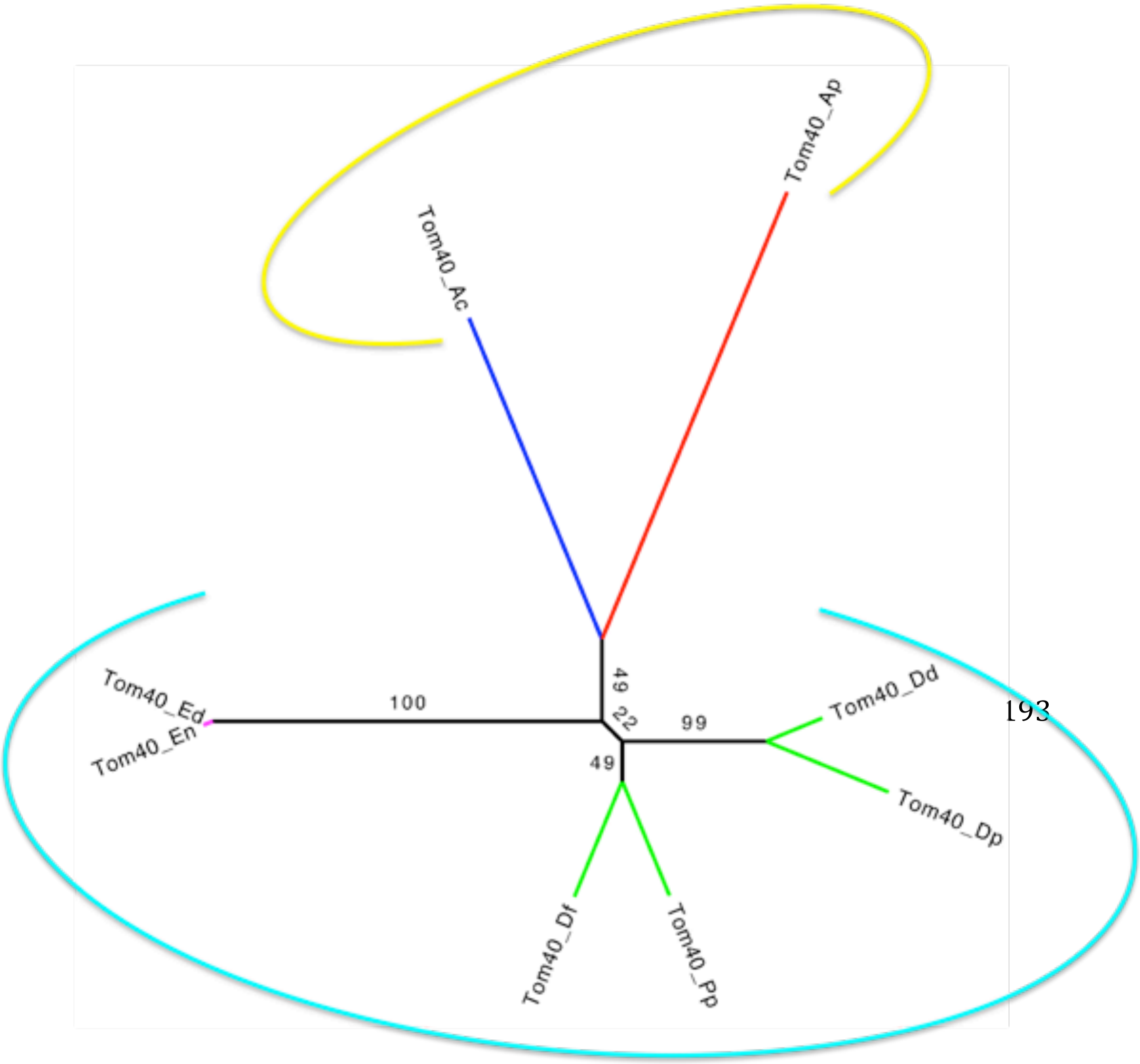

Figure S2B

Tom70

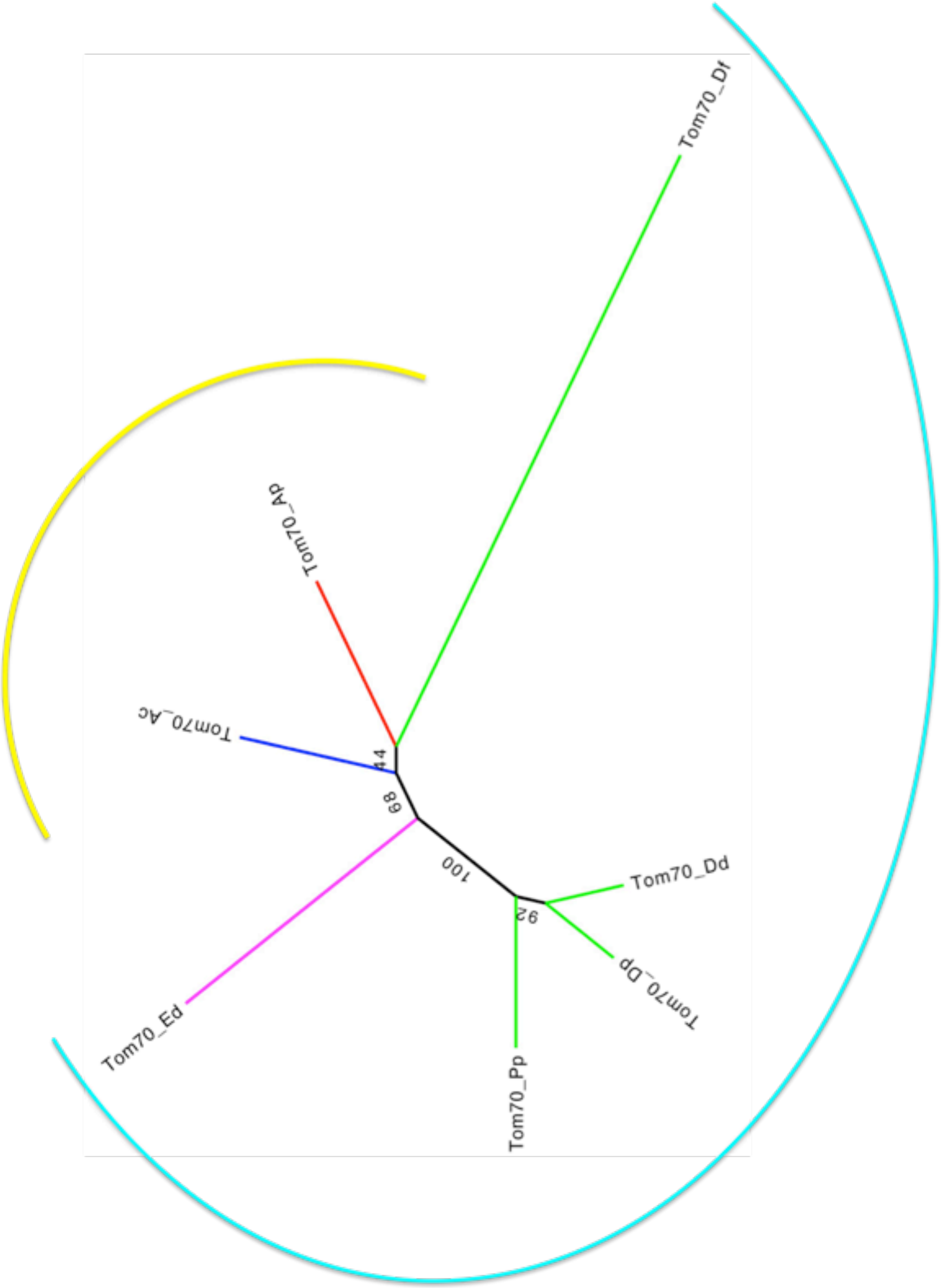

Figure S2C

Metaxin

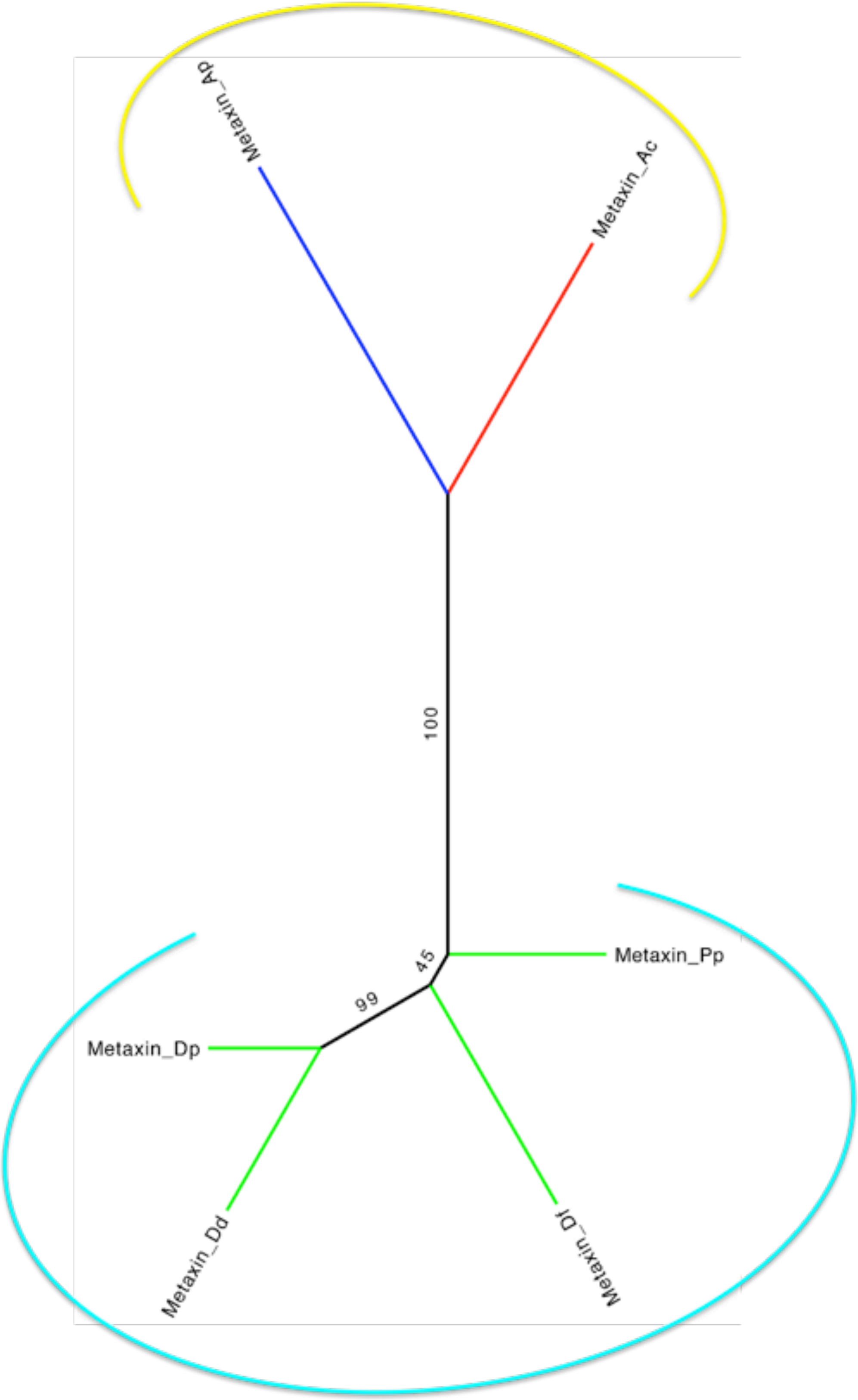

Figure S2D

Tob55/Sam50

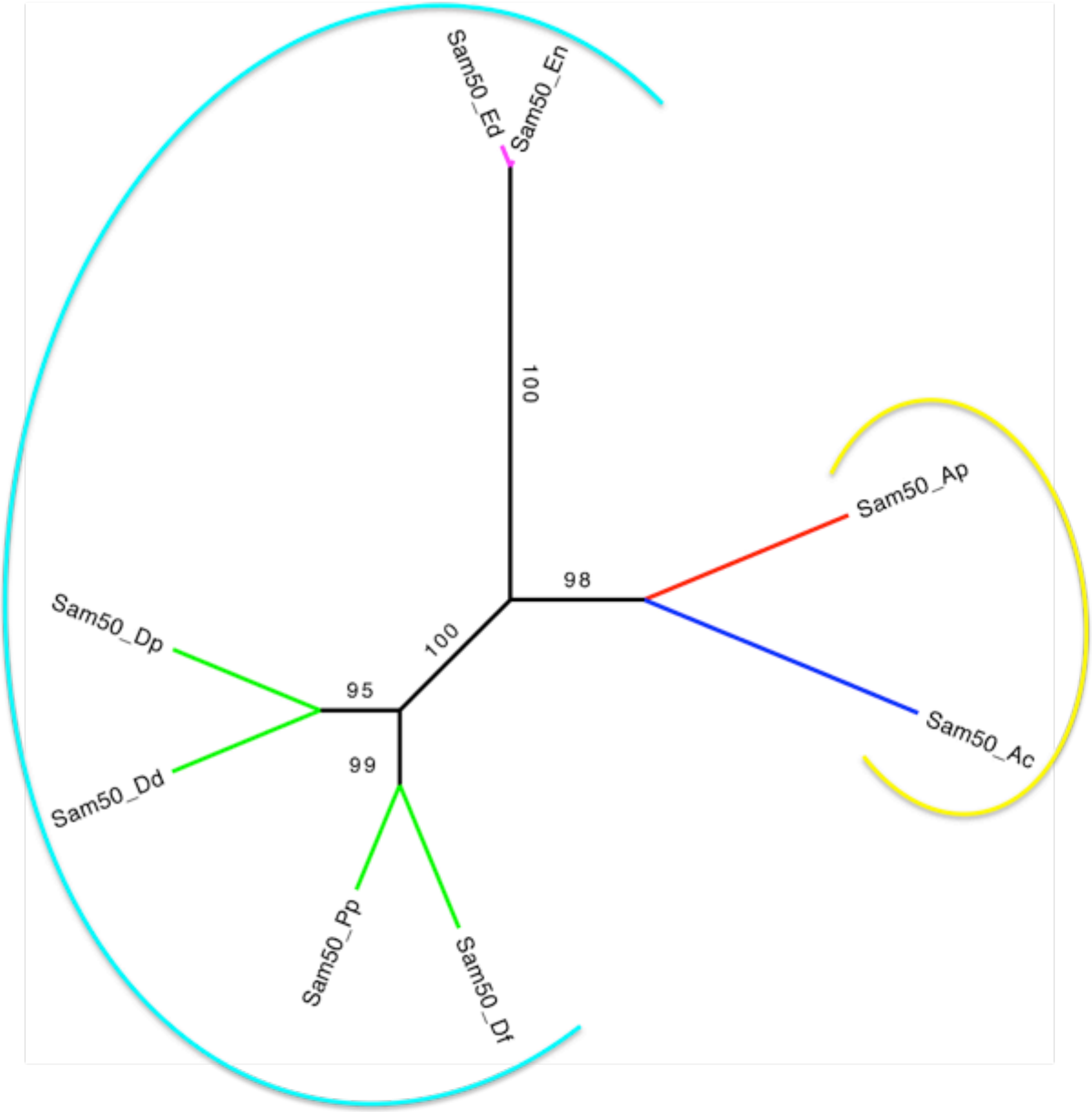

Figure S2E

Mdm10

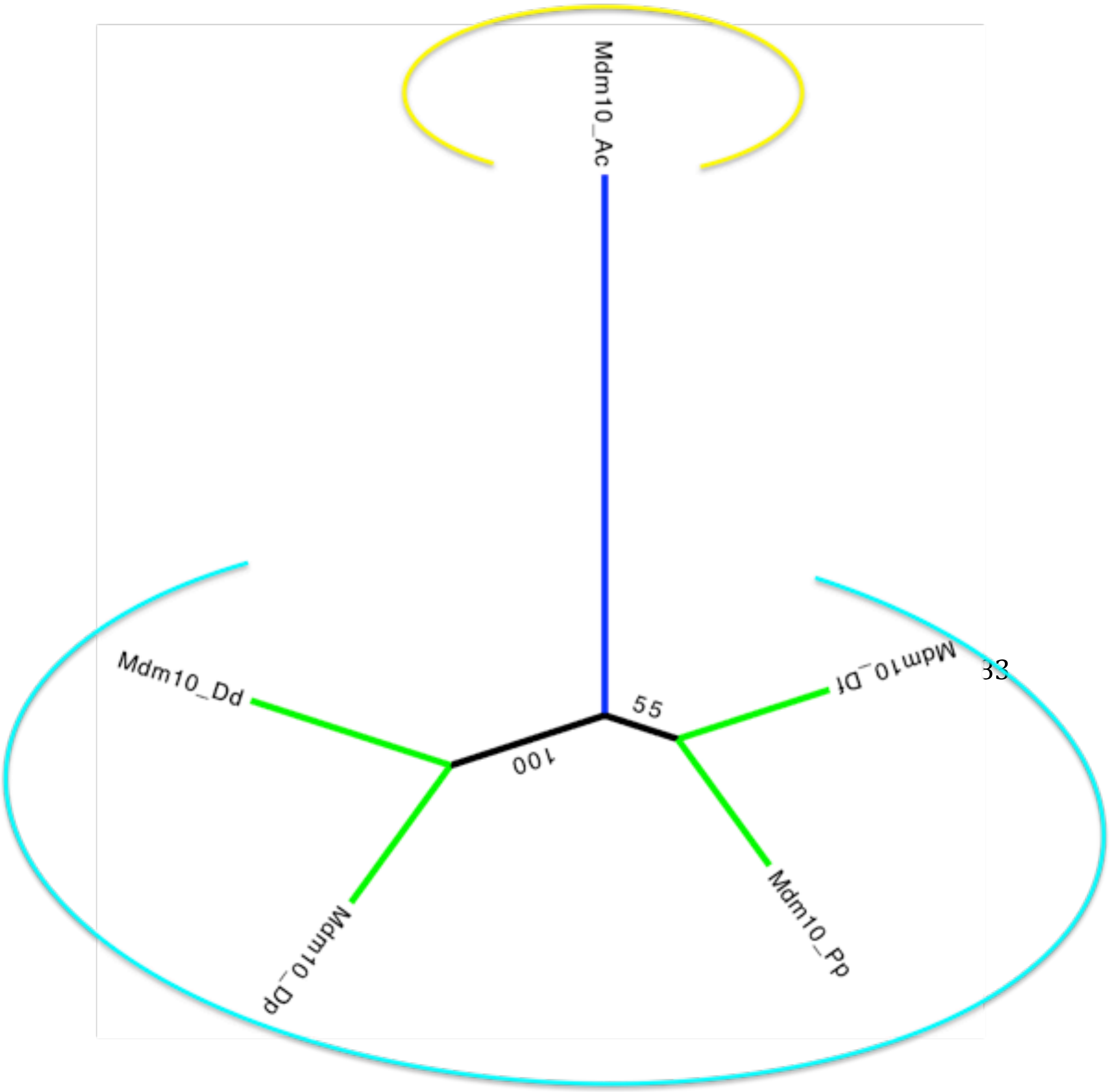

Figure S2F

Mdm12

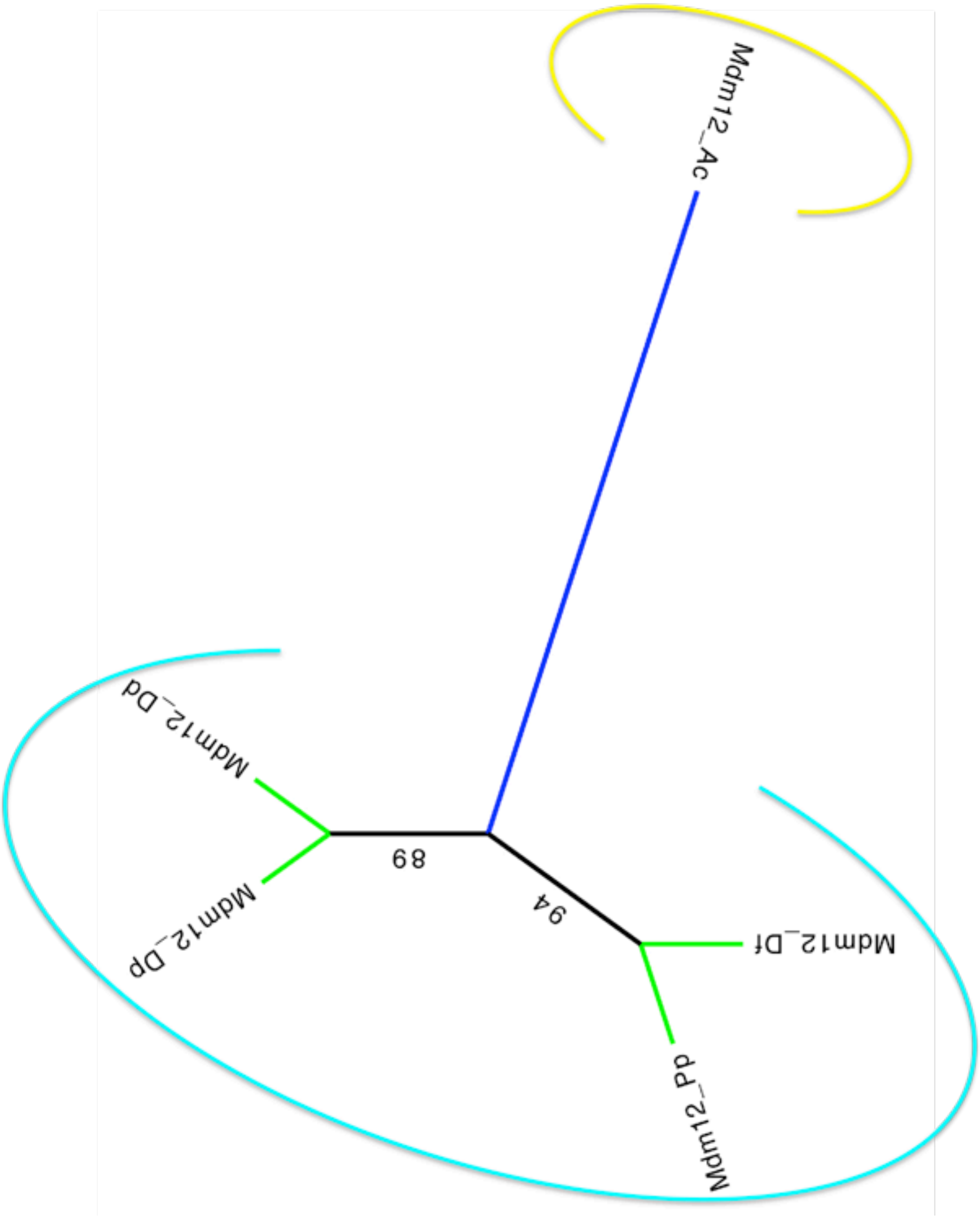

Figure S2G

Mdm34/Mmm2

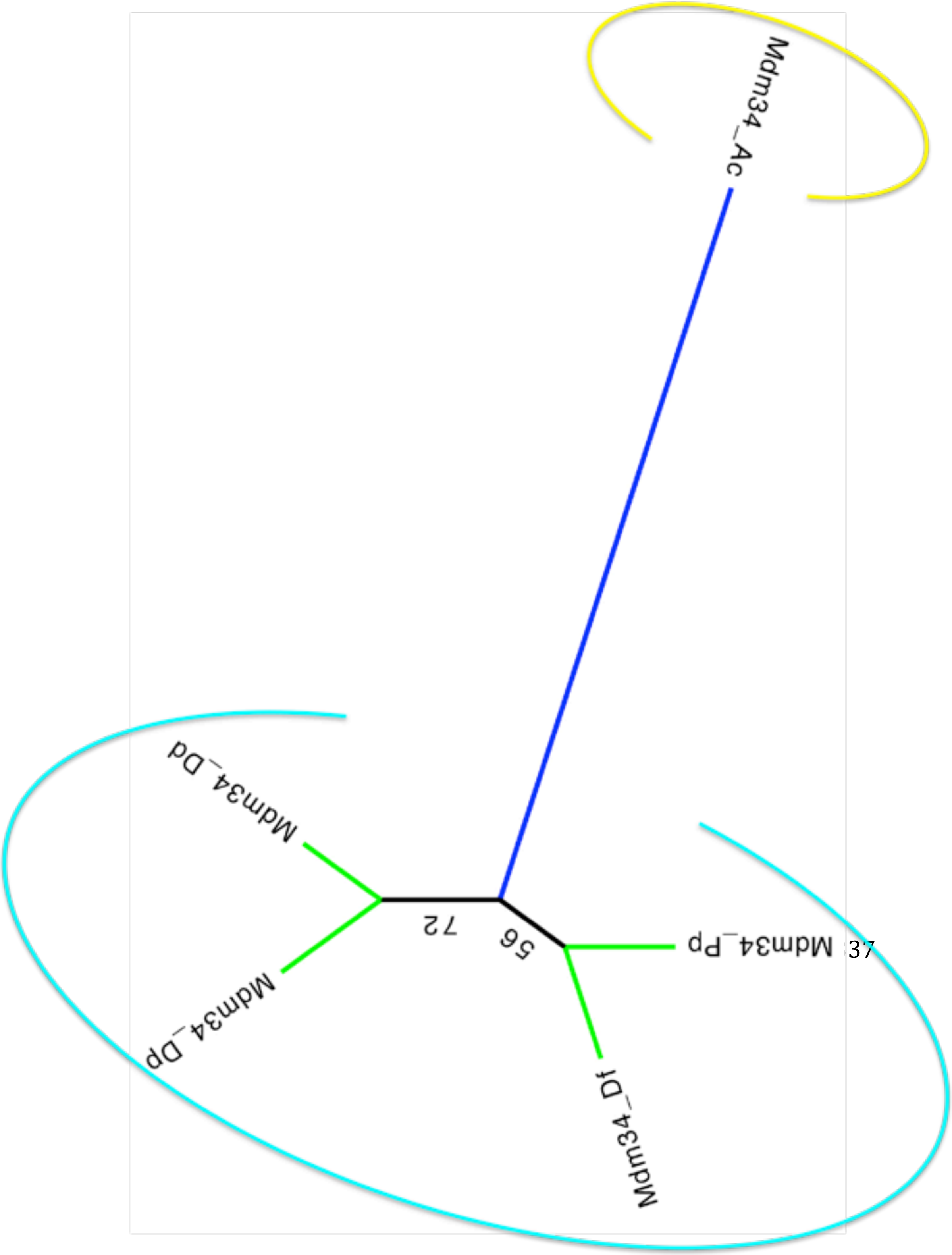

Figure S2H

Mmm1

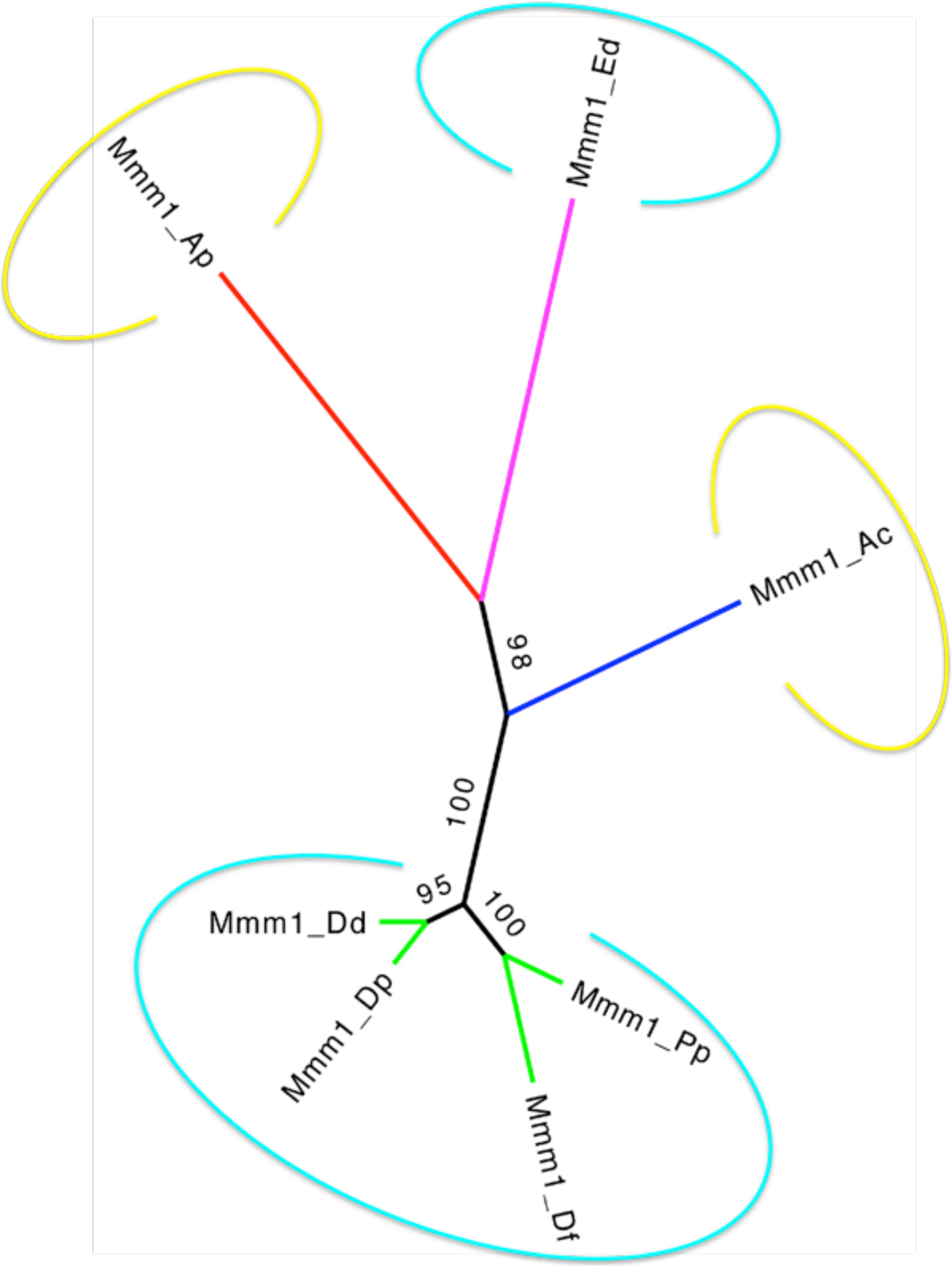

Figure S2I

Gem1

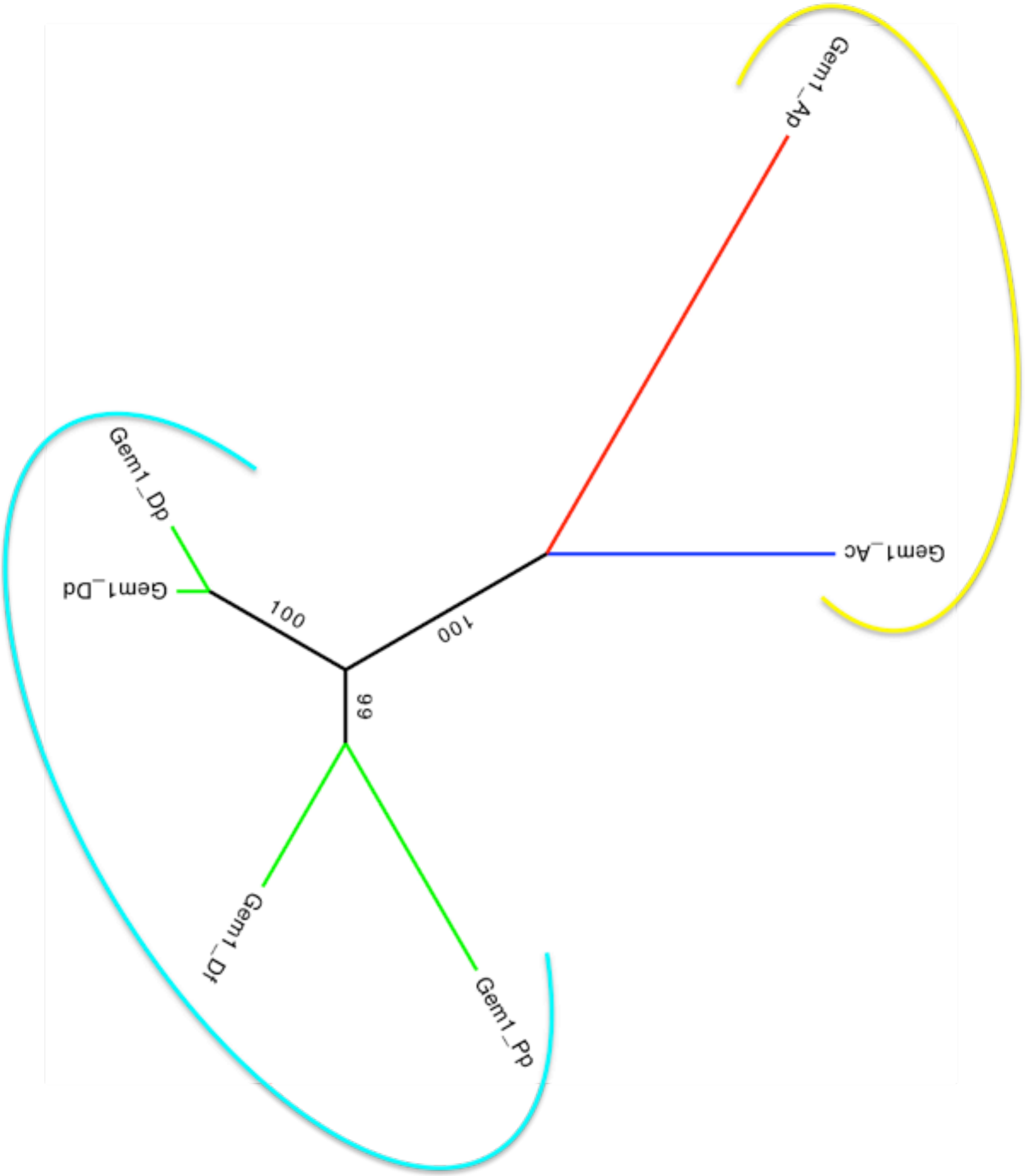

Figure S2J

Figure S3

Graphical representation of the intron – exon gene structure for the identified subunits of the TOM, TOB/SAM and ERMES complexes. Exons are shown in dark blue whereas introns are showed as black thin lines. Light blue color represents alignments of proteins between species. The lengths of introns were neglected. Ac – *A. castellanii*, Dd – *D. discoideum*, Dp – *D. purpureum*, Df – *D. fasciculatum*, Pp – *P. pallidum*, Ed – *E. dispar*, En – *E. nuttalli*

Tom7

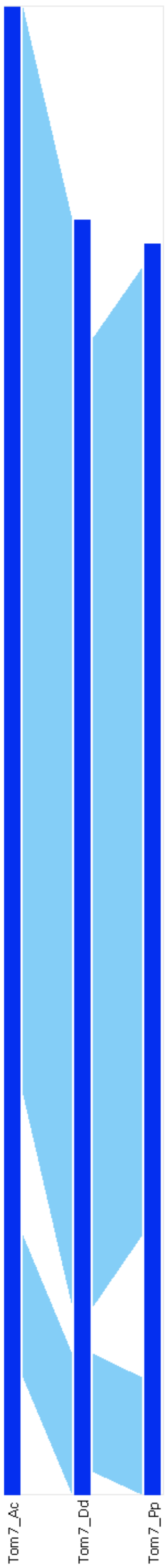

Figure S3A

Tom20

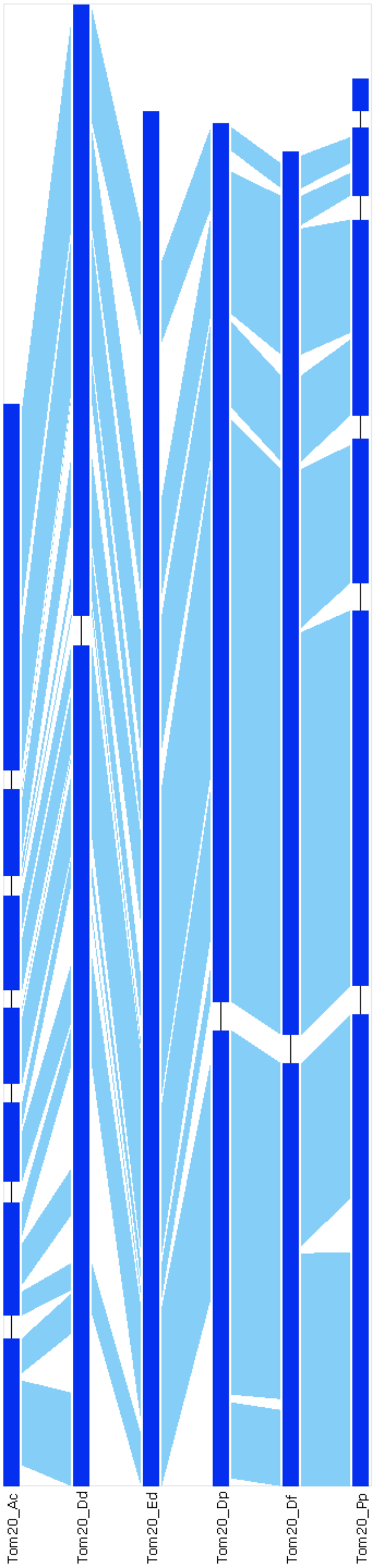

Figure S3B

Tom40

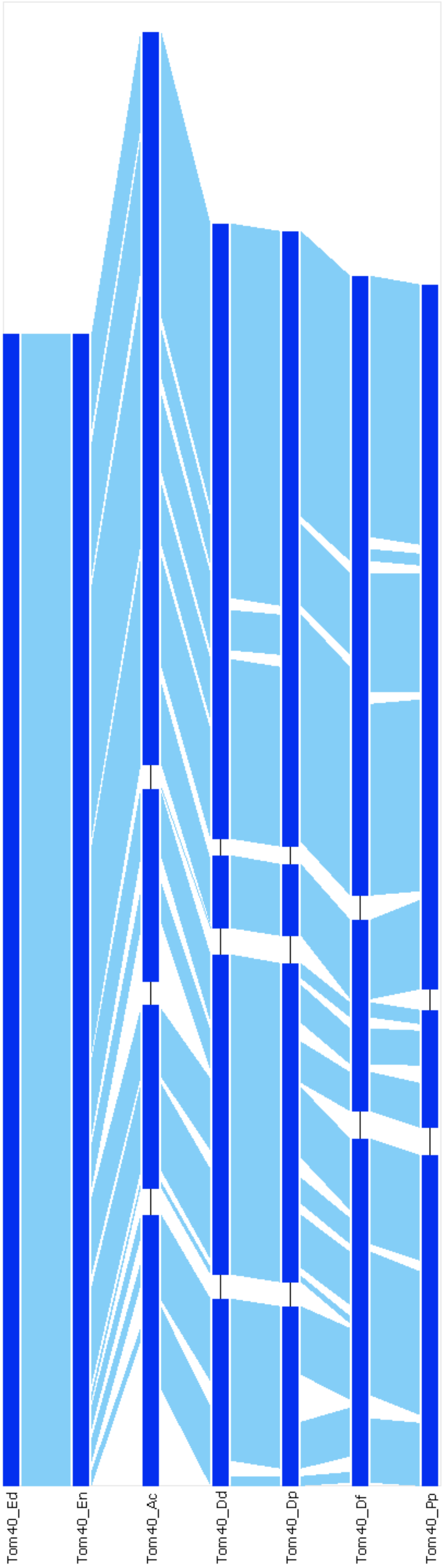

Figure S3C

Tom70

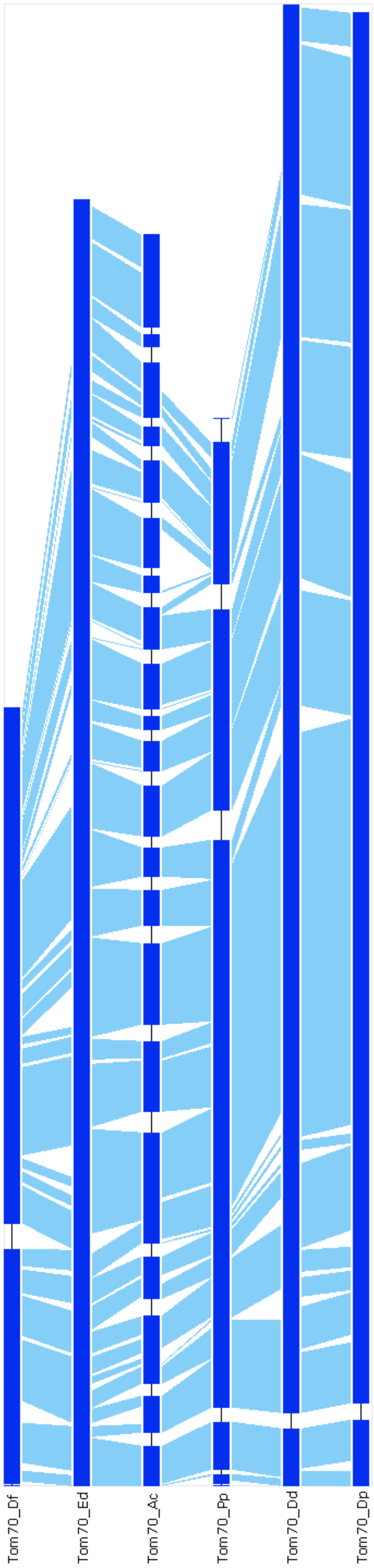

Figure S3D

Metaxin

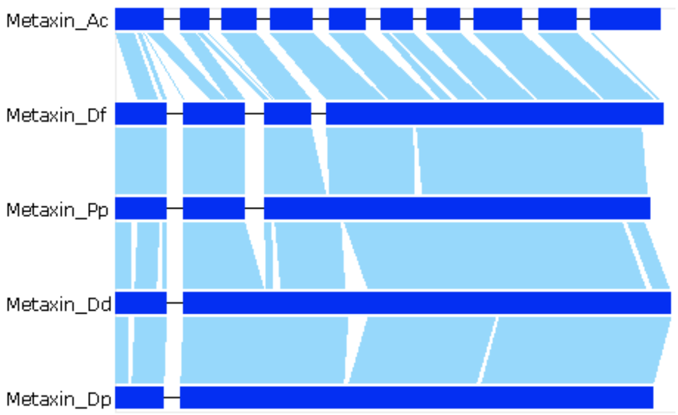

Figure S3E

Tob55/Sam50

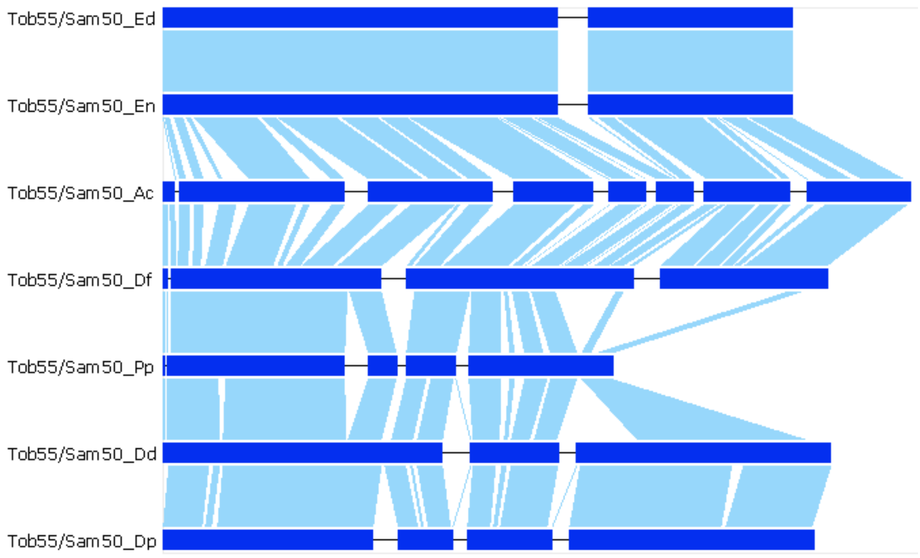

Figure S3F

Mdm10

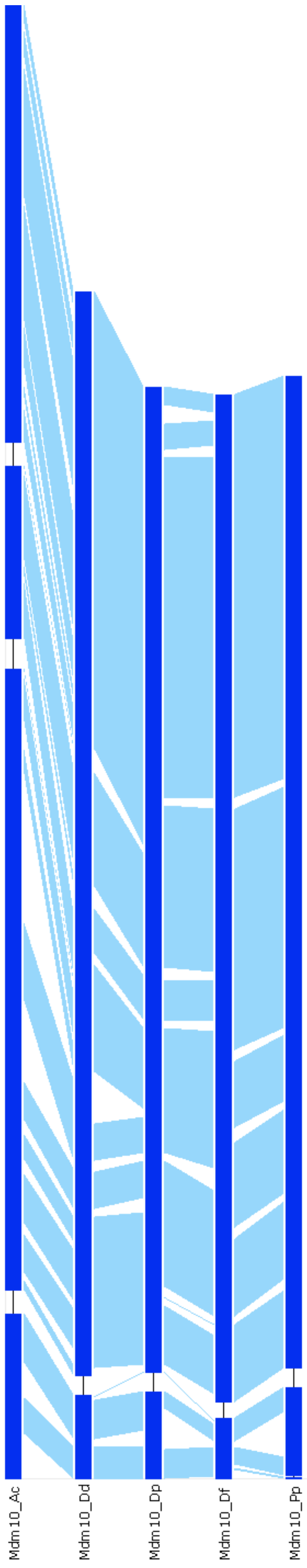

Figure S3G

Mdm12

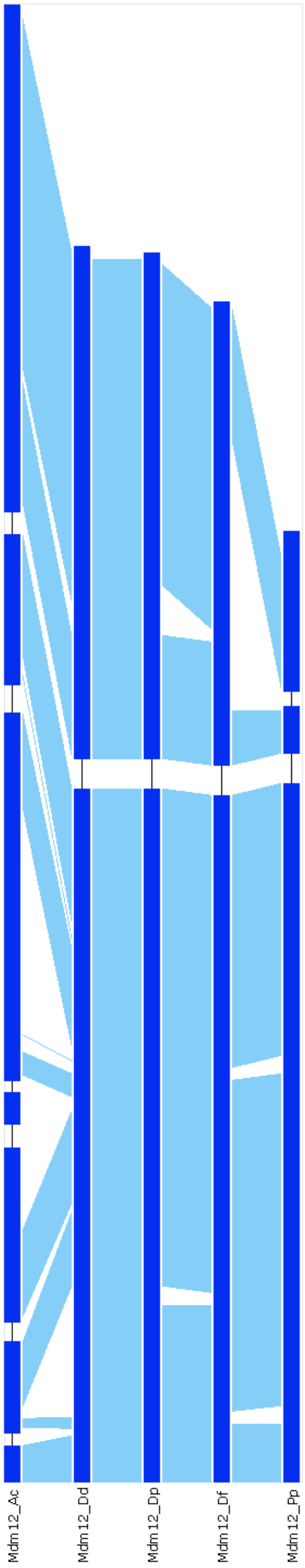

Figure S3H

Mdm34/Mmm2

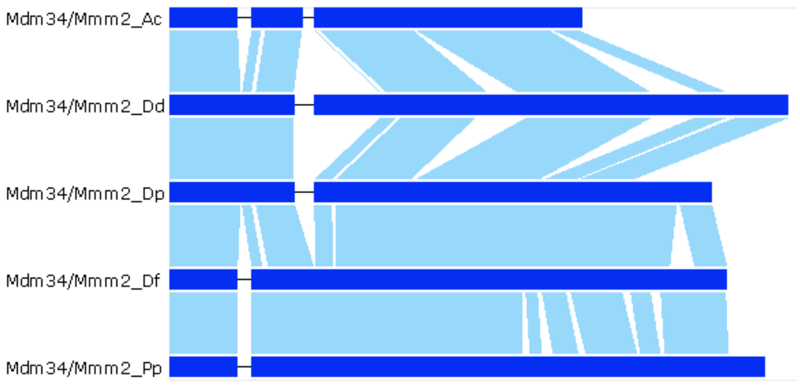

Figure S3I

Mmm1

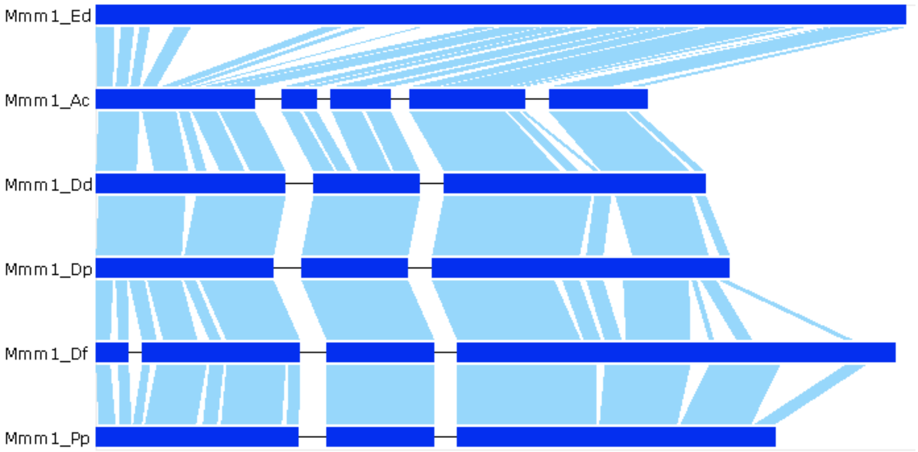

Figure S3J

Gem1

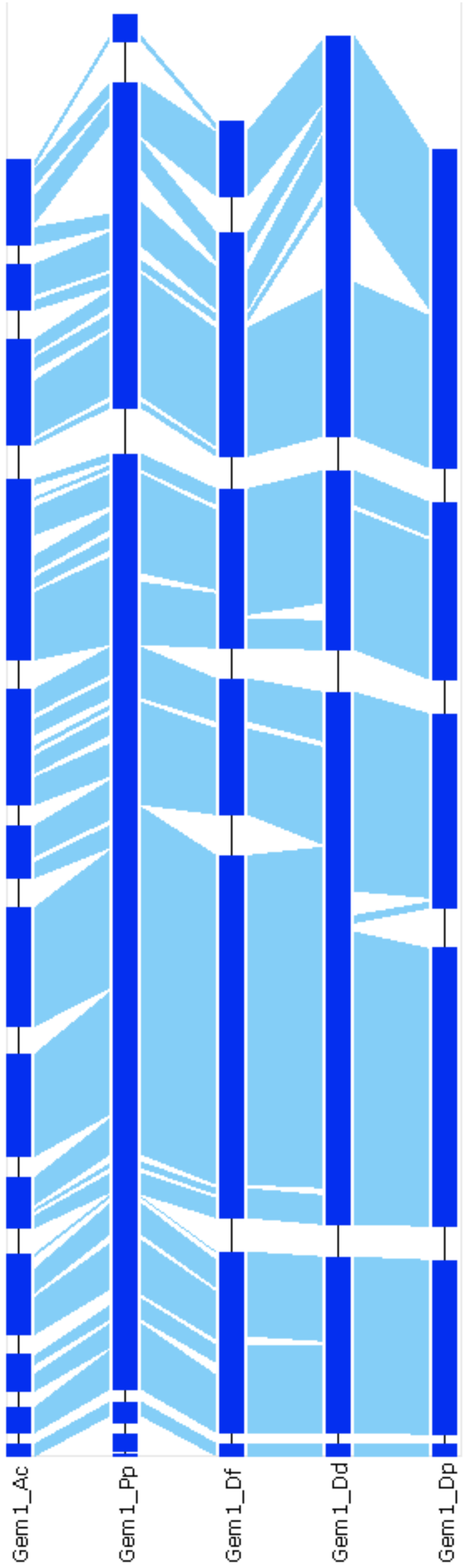

Figure S3K
